# BK channels are indispensable for endothelial function in small pulmonary arteries

**DOI:** 10.1186/s12964-025-02436-0

**Published:** 2025-10-21

**Authors:** Divya Guntur, Dusan Jeremic, Reka Csaki, Oleh Myronenko, Valentina Biasin, Dagmar Kolb, Laura Michalick, Wolfgang M. Kuebler, Peter Enyedi, Horst Olschewski, Andrea Olschewski, Chandran Nagaraj

**Affiliations:** 1 Experimental Anaesthesiology, Department of Anaesthesiology and Intensive Care Medicine, Medical University of Graz, Graz, Austria; 2 Department of Physiology, Faculty of Medicine, Semmelweis University, Budapest, Hungary; 3 Division of Pulmonology, Department of Internal Medicine, Medical University of Graz, Graz, Austria; 4 Division of Physiology and Pathophysiology, Otto Loewi Research Center, Medical University of Graz, Graz, Austria; 5 Core Facility Ultrastructure Analysis, Medical University of Graz, Graz, Austria; 6 Institute for Physiology, Charité - Universitätsmedizin Berlin, Berlin, Germany; 7 DZL (German Centre for Lung Research), partner site Berlin, Berlin, Germany; 8 Faculty of Medicine, Sigmund Freud University, Vienna, Austria; 9 Department of Respiratory Medicine and Critical Care Medicine with Sleep Medicine, Charité - Universitätsmedizin Berlin, Berlin, Germany; 10 BioMedTech, Graz, Austria

**Keywords:** BK channels, Endothelial dysfunction, Hyperpolarization, Pulmonary hypertension, Piezo-1 channels, ROS

## Abstract

**Background:**

Pulmonary hypertension (PH) is a progressive vascular disease that severely compromises quality of life and survival. The pulmonary endothelium plays a pivotal role in vascular homeostasis through complex signalling networks involving ion channels that respond to ionic imbalance (e.g. Na+, K+, Ca2+) and mechanical stimuli (e.g. via Piezo, TRPC, TRPV channels). While large-conductance calcium-activated potassium channels (BK channels), in pulmonary artery smooth muscle cells promote vasorelaxation and attenuate PH, their role in endothelial function is poorly defined. This study investigates the contribution of endothelial BK channels to pulmonary vascular signaling and their potential as therapeutic targets in PH.

**Methods:**

Human lung tissue samples from patients with idiopathic pulmonary arterial hypertension (IPAH) and healthy donors were assessed for BK channel expression by qPCR, Western blot and immunofluorescence staining. BK channel activity in human pulmonary artery endothelial cells was evaluated through patch-clamp recordings. In vivo, BK knockout (BK KO) mice and hypoxia-exposed wild-type mice were used to study endothelial dysfunction and vascular remodeling. Cellular metabolism was analyzed using oxygen consumption rate (OCR) and extracellular acidification rate (ECAR), mitochondrial membrane potential and ROS were assessed by live cell imaging while ex vivo vasoreactivity was assessed via wire myography.

**Results:**

Wild type mice exposed to hypoxia (7 and 28 days) exhibited increased right ventricular systolic pressure (RVSP) and endothelial dysfunction with reduced BK channel function. BK KO mice showed impaired acetylcholine-induced vasodilation of pulmonary arteries, a sign of endothelial dysfunction, similar to mice exposed to hypoxia. BK KO endothelial cells displayed increased mitochondrial respiration, mitochondrial membrane hyperpolarization and increased cellular ROS production. In human PAECs (hPAECs), functional BK channels were identified and in IPAH patients, they were significantly downregulated. Pharmacological BK inhibition in hPAECs resulted in impaired nitric oxide (NO) production and uncontrolled angiogenesis. Furthermore, BK channels colocalized with Piezo-1, and their absence impaired Piezo-1-mediated calcium influx, suggesting a pivotal role in endothelial calcium signaling.

**Conclusions:**

BK channels are integral to pulmonary endothelial signalling, controlling vasodilation, angiogenesis, calcium dynamics, metabolic and oxidative homeostasis. Their impairment causes endothelial dysfunction in PH, and their downregulation in IPAH highlights a novel pathologic mechanism. Restoration of BK channel function may offer a new therapeutic strategy to improve endothelial function and counteract pulmonary vascular remodelling.

**Graphical Abstract:**

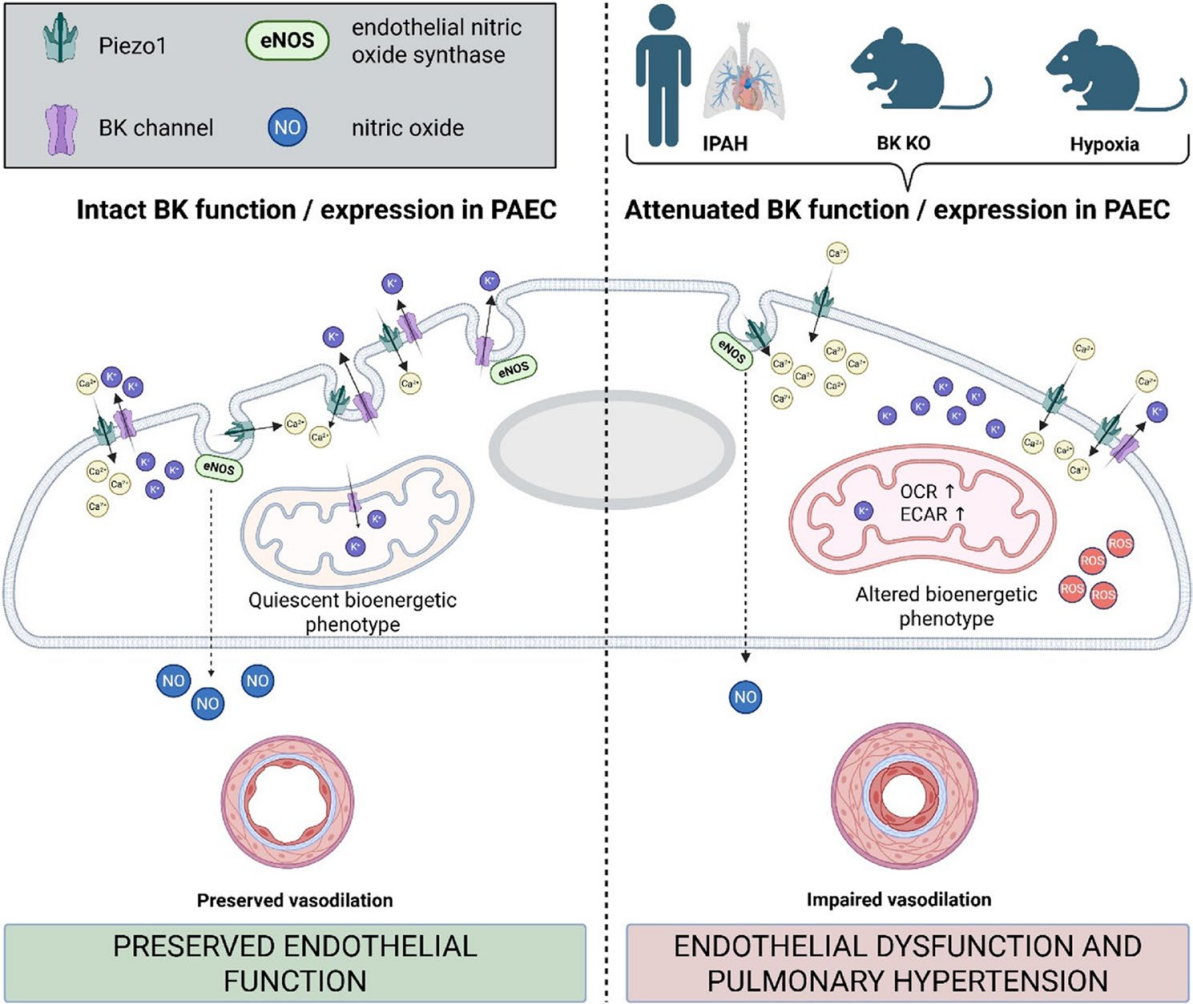

**Supplementary Information:**

The online version contains supplementary material available at 10.1186/s12964-025-02436-0.

## Introduction

Pulmonary hypertension (PH) is a chronic, progressive clinical condition that limits patients’ quality of life and drastically reduces their life expectancy [1–3]. Left-sided heart disease and chronic obstructive pulmonary disease (COPD) represent a significant lifetime risk for PH and drive the overall number of PH patients [4, 5]. This explains why the global prevalence of PH is around 1% of the worldwide population [6] and the steadily increasing prevalence of heart failure and COPD also suggests increasing numbers of PH. Treatment options are only available for a small subset of PH patients with pulmonary arterial hypertension (PAH and chronic thromboembolic pulmonary hypertension (CTEPH)) [7, 8]. The pathogenesis of the disease is multifactorial but pulmonary vascular remodeling is the hallmark of the disease and endothelial dysfunction appears to play a major role [9–13]. However, little is known about the role of ion channels in endothelial dysfunction.

The large conductance calcium-activated potassium channel (BK) is a critical controller of vascular tone and architecture [14]. BK are activated by changes in both voltage and intracellular calcium concentration ([Ca^2+^]_i_). As a result of BK activity, the cells tend to repolarize/hyperpolarize. The apparent Ca^2+^- and/or voltage-sensitivity is directly regulated via various kinases including cAMP-dependent protein kinase (PKA), cGMP-dependent (PKG). In pulmonary and systemic arterial smooth muscle cells, BK channels mediate the effects of prostacyclin receptor agonists and NO [15]. This, along with KCNK3/TASK-1 channel activity [16] represents the most important mechanism of prostacyclin-induced vasodilation. In addition, dysregulated expression or activity of BK has been implicated in various cardiovascular diseases and vascular aging, suggesting that BK also plays an important role in vascular remodeling [17–20].

In this study, we investigated the expression and functional role of BK channels in the pulmonary artery endothelium of IPAH patients and their controls, and in a mouse model of PH. While BK channels have been extensively studied in smooth muscle cells (see [21] for more details), their expression and physiological significance in endothelial cells is less well characterized and subject to debate [22, 23]. Our findings demonstrate endothelial BK channel expression and highlight their contribution to pulmonary vascular signaling and homeostasis. Elucidation of BK channel-mediated endothelial signaling may provide new insights into pulmonary vascular regulation that foster the development of targeted, lung specific interventions to restore endothelial function in PH disease.

## Methods

### Human lung samples

Human cells and tissues used at Stanford were obtained through the Pulmonary Hypertension Breakthrough Initiative (PHBI), funded by the NIH (R24 HL123767) and the Cardiovascular Medical Research and Education Fund (CMREF; UL 1RR024986).

Human lung tissue samples used at the Medical University of Graz were obtained from patients with idiopathic pulmonary arterial hypertension (IPAH) who underwent lung transplantation at the Medical University of Vienna, Department of Thoracic Surgery or served as lung donors. The study protocol for obtaining the human tissue at the Medical University of Vienna was approved by the Institutional Review Board (approval number 976/2010) and written informed consent was obtained from each participant prior to transplantation. The demographics of these samples have been previously described [24, 25]. The sole inclusion criterion was end-stage idiopathic pulmonary arterial hypertension (IPAH), with elevated mean pulmonary arterial pressure (mPAP) of ≥ 25 mmHg. Control tissues were provided from donor lungs that met al.l criteria for transplantation but were downsized to fit recipients of smaller stature and were not used for the actual transplant. The diagnosis was confirmed by experienced pathologists and pulmonologists who reviewed the clinical data and reviewed the chest CT scans and right heart catheterization results. Neither the lung recipients nor the donors were diagnosed with any chronic lung disease with the exception of pulmonary arterial hypertension.

In addition, human control PAEC were obtained from Lonza (Basel, Switzerland). Information on the gender of the donors and patients, as well as the tissues used, is provided in Supplementary Table 1.

### Animal studies

All experimental procedures were performed with approval by the local authorities in accordance with national regulations (Austrian Ministry of Education, Science and Culture, 2022 − 0.619.415). The animals were housed under standard conditions, including a 12-hour light/dark cycle, controlled room temperature and unrestricted access to food and water.

For the chronic hypoxia-induced pulmonary hypertension (PH) model, male C57BL/6J mice (aged 11–13 weeks) were obtained from Charles River Laboratories and randomly assigned to either normoxic or hypoxic groups. Control animals were kept under normobaric, normoxic conditions (21% O₂), while hypoxic mice were housed in normobaric, hypoxic chambers (10% O₂) for either 7 or 28 days. Oxygen levels were continuously monitored and regulated by an automated OxyCycler system (BioSpherix, Lacona, NY, USA) with nitrogen gas buffering to maintain hypoxia. Chambers were briefly opened twice weekly for food and bedding changes.

Endpoint measurements were performed at days 7 (*n* = 5 normoxia and *n* = 5 hypoxia) and 28 (*n* = 5 normoxia and *n* = 5 hypoxia) at which point the mice were 12–16 weeks old. Each hypoxic mouse was directly compared to an age-matched normoxic control at the time of endpoint measurements. Hemodynamic measurements were performed in mice anesthetized with 2% isoflurane in oxygen throughout the procedure, using the closed-chest technique via a small incision in the submandibular region. Body temperature was maintained at 38 ± 1 °C, while ECG recordings were used to monitor heart rate stability. Right ventricular pressure was measured using a 1.4-Fr Millar catheter (SPR-671; Millar, Houston, TX, USA) inserted via the right jugular vein. Following hemodynamic measurements, mice were euthanized, lungs were harvested for pulmonary artery (PA) isolation, and vascular reactivity studies were performed. Hearts were collected and weighed for calculation of Fulton-Index.

### Evaluation of pulmonary arterial vasoreactivity ex vivo

Mice were 12–16 weeks old when they were euthanized by cervical dislocation. Pulmonary arteries obtained from BK WT and KO mice, as well as C57BL/6J mice kept either under hypoxic or normoxic conditions were investigated. Lungs were harvested to isolate intrapulmonary arteries (PAs), which were then mounted on a wire myograph (Danish Myo Technology 620 M, Aarhus, Denmark) using tungsten wires. Vessels were equilibrated for 30 min in physiological saline solution (PSS; pH 7.4), fully oxygenated and maintained at 37 °C. Baseline tension was gradually adjusted to 2 mN and stabilized for an additional 30 min [25–27].

#### Vessel quality control

Vessel viability was assessed by three consecutive 15-minute depolarizations with potassium-rich PSS (KPSS; 120 mM KCl substituted for NaCl to maintain isotonicity). Vessels with a mean response of < 2 mN were excluded. Isometric tension was recorded using force transducers connected to the myograph system (LabChart Pro v8.1.25, ADInstrument, Oxford, UK). Following KPSS exposure, vessels were washed with PSS to ensure complete removal of KPSS and were used for endothelial integrity testing.

#### Endothelial integrity

Endothelial integrity was evaluated by inducing vasoconstriction with 1 µM phenylephrine (PE), followed by the addition of 10 µM acetylcholine (Ach) to assess endothelium-dependent relaxation. Vessels showing < 50% relaxation in response to Ach were excluded. Vessels were washed again and used in experimental protocols.

For studies requiring endothelial denudation (negative controls), the endothelial cell layer was mechanically removed by gently rubbing the luminal surface with a strand of human hair prior to mounting. As expected, all denuded vessels exhibited < 50% vasodilatory response to Ach.

#### Experimental protocol

From each mouse, four intrapulmonary PAs were mounted in separate myograph chamber units. Only vessels that passed both the vessel viability and endothelial integrity tests were included in subsequent experiments. Vasorelaxation was expressed as percentage of the maximal vasoconstrictor-induced response.

### Cell culture and handling

#### Human pulmonary artery endothelial cells (PAECs)

PAECs from donors and IPAH patients were isolated and harvested from PAs less than 2 mm in diameter. The endothelial layer was treated with a mixture of collagenase, DNase and dispase in Hank’s Balanced Salt Solution (HBSS) at room temperature. After enzymatic digestion, the cell suspension was collected, resuspended in complete endothelial medium (Lonza, Basel, Switzerland and ScienCell, CA, USA) and cultured in gelatin-coated T25 flasks at 37 °C and 5% CO_2_. Once the cells reached 70–80% confluence, they were trypsinized and subjected to three rounds of CD31-selective magnetically activated cell sorting to enrich the endothelial cell population. Cell identity was verified by morphology and marker analysis (smooth muscle actin, fibronectin, vimentin, von Willebrand factor, smooth muscle myosin heavy chain and CD31). Extra PAECs were cryopreserved in endothelial cell complete medium with 10% foetal calf serum (FCS) and 10% dimethyl sulphoxide (DMSO) and stored in Liquid nitrogen for later use. The experiments were performed with cells from passages 3 to 9. Human PAECs (hPAECs), whether purchased (Lonza, Basel, Switzerland or PromoCell, St. Louis MO, USA) or isolated as described, were cultured in gelatin-coated flasks (0.1% gelatin solution in PBS) with endothelial cell complete medium, containing antibiotics (penicillin and streptomycin).

#### Isolation of mouse lung endothelial cells

Mouse lung was placed on a Perti dish and cut into pieces as small as possible with a scalpel. The tissue was then placed into a 50 mL centrifuge tube containing 4 mL of digestion solution: 3 mg/ml collagenase I (Gibco, Carlsbad, CA, USA) and 0.1 mg/ml DNase (Serva electrophoresis, Heidelberg, Germany) in DMEM F12 basal medium. The falcon was then placed on a shaker at 37 °C for 45 min for tissue digestion. The digested tissue suspension was then filtered through a 100 μm cell sieve. To stop digestion, an equal volume of DMEM F12 complete medium with fetal calf serum (FCS) was added. The suspension was centrifuged at 1200 rpm and 4 °C for 8 min. The supernatant was discarded and the pellet was resuspended in CD31 coated dynabeads. For a pair of mouse lungs, 15µL dynabeads (Invitrogen, Waltham, MA, USA) were coated with 1.5µL CD31 antibody (BD Pharmigen, Franklin Lakes, NJ, USA) overnight on a shaker at 4 °C and washed the following day with sterile PBS to remove any unbound antibody, and resuspended in 100µL PBS, in a microcentrifuge tube. This tube was placed on a shaker for 15 min at room temperature. The tube containing the beads was then placed on a magnetic stand and the supernatant was discarded. The tube was removed from the magnetic stand, the beads were re-suspended in 1 mL medium and the procedure was repeated approximately six times to remove all non-CD31-specific cells. After the last wash, the beads were resuspended in endothelial growth medium (Lonza EBM with microvascular endothelial growth supplements) and seeded on a gelatin-coated T25 cell culture flask. After 48 h, the unattached cells were removed by washing once with PBS, and fresh medium was then added every day until the cells were confluent. For the experiments, the result of isolation of ECs from the lungs of 2–3 mice were pooled.

### Detection of mRNA expression levels

hPAECs were cultured until they reached confluence. RNA was isolated either by Trizol reagent or by an RNA isolation kit (Zymo research, Irvine, CA, USA) according to the manufacturer’s protocol. Complementary DNA (cDNA) synthesis was performed using a cDNA synthesis kit (iScript, Bio-Rad, Hercules, CA, USA or Takara, Shiga, Japan) according to the manufacturer’s instructions. The expression levels of the target genes were then analysed by quantitative PCR (qPCR). The qPCR was performed using Applied Biosystems PowerTrack SYBR Green Master Mix (ThermoFisher Scientific, Waltham, MA, USA) in a standard thermocycler. The SYBR Green dye intercalates with the double-stranded DNA during amplification, enabling quantification of gene expression based on fluorescence intensity.

The used primer sequences are given below (5’ to 3’).

### SiRNA Silencing

hPAECs were plated in 6-well plates. After 24 h, the medium was removed and 900 µL was added per well. The jetPRIME treatment solution was prepared by adding jetPRIME buffer (96.75 µL/well) (Polyplus, New York city, NY, USA) and siRNA/siControl (1.25 µL/well of 20 µM) (Smartpool, Dharmacon, Horizon Discovery Limited, Lafayette, CO, USA). The mixture was shaken and centrifuged. To this, 2 µL/well of jetPRIME reagent was added, then vortexed, centrifuged and incubated for 15 min at room temperature. This is the treatment solution. The treatment solution (100 µL) was added dropwise per well containing 900µL medium and the plate was placed back in incubator at 37 °C. The medium was changed 24 h after treatment. The cells for RNA isolation were lysed after 48 h of treatment.

### Protein isolation and Western blot analysis

Once the seeded hPAECs on the plates reached confluency, they were washed twice with Dulbecco’s phosphate buffered saline (DPBS) to remove any residual media or non-adherent cells. Subsequently, 200 µL of RIPA with protease and phosphatase inhibitors was added to each well to lyse the cells and extract proteins. The cell lysates were collected and centrifuged at 10,000 g for 15 min at 4 °C to separate the supernatant containing the soluble proteins. The supernatant was transferred to fresh tubes for further analysis. The total protein concentration of each sample was determined using the bicinchoninic acid (BCA) assay. The protein samples were prepared by mixing with 4X loading dye containing betamercaptoethanol in a 1:4 ratio and heating at 95 °C for 5 min to denature the proteins. The proteins (20 µg per sample) were loaded onto a 10% agarose gel and electrophoresed with Tris-glycine running buffer to separate the proteins by molecular weight. After gel electrophoresis, the proteins were transferred to a nitrocellulose membrane using 1X transfer buffer with 10% methanol to ensure efficient transfer of proteins from the gel to the membrane. An alternative procedure included the use of 1% sodium dodecyl sulphate (SDS) in Tris-HCl buffer instead of RIPA, TCEP instead of betamercaptoethanol, a pre-made 4–6% Bis-Tris gel (Thermofisher Scientific, Waltham, MA, USA) instead of a 10% agarose gel, MOPS running buffer (Thermofisher Scientific, Waltham, MA, USA), a polyvinylidene difluoride (PVDF) membrane, Immobilon-E (Merck, Germany) instead of a nitrocellulose membrane and Nupage transfer buffer (Thermofisher Scientific, Waltham, MA, USA). After transfer, the membranes were blocked with 5% bovine serum albumin (BSA) to prevent non-specific antibody binding. The membranes were then incubated overnight with the primary antibodies, specific for the proteins of interest (anti-BK: APC-021, Alomone, Isreal and L6/60, NeuroMab UC Davis, CA, USA, anti-PIEZO1: 15939-1-AP, Proteintech, Rosemont, IL, USA, anti-caveolin1: D46G3 #3267, Cell signaling, Danvers, MA, USA, anti- GAPDH: ab9485, Abcam, Cambridge, UK, anti-B-Actin: sc4778, Santa Cruz, Dallas, TX, USA, anti- alpha tubulin: 2125 S, Cell signaling, Danvers, MA, USA). After overnight incubation with the primary antibodies, the membranes were washed three times with TBST (Tris-buffered saline with 0.1% Tween 20) for 5 min each time to remove unbound antibodies. The membranes were then incubated with the corresponding secondary antibodies for 1 h at room temperature. After washing, the membranes were developed with enhanced chemiluminescence (ECL) or ECL Femto Substrate (Bio-Rad, Hercules, CA, USA) to visualize the protein bands. The densitometries were calculated using Image lab software (Bio-Rad, Hercules, CA, USA). Signal density was normalized to either β-actin, α- tubulin or GAPDH as indicated.

### Proximity ligation assay

hPAECs were plated on chamber slides and left overnight. The next day, the wells were washed with DPBS and fixed with 4% paraformaldehyde (PFA) for 15 min. The PFA was removed and the wells were washed three times with DPBS and stored at 4 °C until staining. DPBS was removed and 5% donkey serum without Triton was used as blocking buffer. The slide was incubated for 1 h at room temperature. Primary antibodies against BK (L6/60, NeuroMab UC Davis, CA, USA) mouse antibody and Piezo-1 (15939, Proteintech, Rosemont, IL, USA, USA) rabbit antibody were added at a dilution of 1:200 and incubated overnight at 4 °C. The primary antibodies were removed and the wells were then washed with TBST. For the further steps, the Duolink PLA kit (Merck, Germany) was used according to the manufacturer’s protocol. When the two target proteins are in close proximity, a long single-stranded DNA product with fluorescence is formed, which was then detected with a fluorescence microscope.

### Immunofluorescence staining

hPAECs or mouse endothelial cells were plated on chamber slides and incubated at 37 °C for 24 h. Cells were then quickly washed with 1X PBS and fixed with 4% formalin for 20 min at room temperature. For mitochondrial staining, cells were washed with 1X PBS and incubated with a mitochondrial marker for 30 min (MitoTracker Red CMXRos, M7512, Thermo Fisher Scientific, Waltham, MA, USA) prior to 4% formalin fixation. Formalin was removed and slides were washed three times with 1X PBS and stored in PBS at 4 °C until use. Blocking buffer, 3% BSA with 0.1% Triton X-100 was added on the slides and stored at room temperature for 1 h. Human lung sections containing PAs which were paraffin embedded were deparaffinized, rehydrated and treated with heat-induced antigen retrieval in pH 6 buffer. Blocking buffer with 10% BSA was added on the tissue slide and incubated at room temperature for 1 h. Subsequent staining steps for both cells and tissue slides remained the same after this. 200 µL/well of the primary antibody (anti-BK: APC-021, Alomone, Isreal, anti- vWf (anti- von Willebrand factor): A0082, Dako, Glosturp, Denmark) diluted in blocking buffer was added and incubated overnight at 4 °C. The slides were then washed three times with PBS and a fluorophore-conjugated secondary antibody diluted in blocking buffer was added and incubated for one hour at room temperature. The secondary antibody solution was removed and the slides were washed three times with PBS. 10uL of Vectashield mounting medium with DAPI (Vector Laboratories, Peterborough, UK) was added per well, the coverslip was placed on the slide and stored in the dark at 4 °C until Images were taken with the Nikon A1 + confocal microscope or Zeiss LMS 510 META. Images of chambers with duplicates without primary antibodies were used as negative controls.

### Nitric oxide assay

hPAECs were plated in gelatin-coated black 96-well plates and starved in Ringer’s solution for 1 h. Cells were then loaded with 10 µM 4-amino-5-methylamino-2′,7′-difluorofluorescein diacetate (DAF-FM, Thermo Fisher Scientific, Waltham, MA, USA) for 30 min at 37 °C. After loading, the cells were washed twice with Ringer’s solution and treated with drugs dissolved in Ringer’s solution, followed by incubation at 37 °C for 10 min. Cells were then stimulated with 5 µM acetylcholine (Ach) and fluorescence was immediately measured using a CLARIOstar Plus plate reader (BMG Labtech, Ortenberg, Germany) with excitation/emission wavelengths set to 495/515 nm.

### Assessment of ion channel activity

Whole-cell patch-clamp experiments were performed as previously described (Lengyel et al., 2019) using human Lonza PAECs. The bath solution contained (in mM): KCl 4, NaCl 140, CaCl_2_ 2, MgCl_2_ 1, HEPES 10 (pH 7.4, adjusted by NaOH). The pipette solution contained (in mM): KCl 135, NaCl 10, EGTA 1, CaCl_2_ 0.379, HEPES 10 (pH 7.2, adjusted by NaOH), with free calcium concentration set to 100 nM. Whole-cell currents were recorded in voltage-clamp mode, with a holding potential of −60 mV. BK current was evoked by applying 200-ms voltage steps ranging from − 60 mV to + 100 mV in 20 mV increments, currents were measured at the end of each voltage step. After measuring the control current traces, paxilline (2 µM) or iberiotoxin (100 nM) were applied by direct perfusion for 2–3 min, before measuring the inhibited current. The effect of the drugs was tested on a separate set of cells. Capacitive transients and series resistance were carefully compensated to ensure accurate current measurements and minimize artifacts. Data were analyzed using pCLAMP 10.7 software (Molecular Devices, Sunnyvale, CA, USA), and all currents were leak-subtracted.

### Angiogenesis assay

Matrigel tube formation test kit (Merck Millipore, Burlington, MA, USA) was used. ECMatrix was diluted on ice according to the manufacturer’s instructions and 50 µL was applied to a 96-well tissue culture plate. The ECMatrix was then incubated at 37.0 °C to polymerise and solidify. This provides the basis for the formation of tubes by the cells. 50 × 10^3 hPAECs were then seeded on the surface of the gel and returned to the incubator. The media contained paxilline and iberiotoxin as treatment, while the control wells were left untreated. The wells in which the cells grew on the ECM matrix and formed tubes were imaged every hour for 6 h using an Olympus CKX41 light microscope. The images were then analysed with the macro angiogenesis analyzer of the ImageJ software to calculate the values of the network parameters.

### Assessment of cytosolic Ca^2+^

Cells were incubated with 2 µM Fura-2-acetoxymethyl ester (Fura-2AM) at 37 °C for 45 min. A single glass coverslip was placed on the stage of a Zeiss 200 M inverted epifluorescence microscope with a PolyChrome V monochromator light source (Till Photonics, Kaufbeuren, Germany) in a sealed, temperature-controlled RC-21B imaging chamber (Warner Instruments, Hamden, CT, USA). Fluorescence images were acquired every 3 s with alternating excitation wavelengths of 340 nm and 380 nm, with emission at 510 nm recorded via an air-cooled Andor Ixon camera (Andor Technology, Belfast, Ireland). The background fluorescence of each coverslip was measured and subtracted before performing the calculations. Images were stored and analyzed offline using TillVision software (Till Photonics, Germany).

At 75 images when the baseline was stable, cells were treated with Yoda1. At the end of each experiment, the maximum and minimum ratio values were determined by treating the cells with 5 µM ionomycin to determine the maximum ratio, followed by chelation of total free Ca²⁺ with 20 mM EGTA to determine the minimum ratio. Cells that did not respond to ionomycin were excluded from the analysis.

### Ultrastructure analysis

Mouse PAs were fixed for 3 h in 2.5% (w/v) glutaraldehyde and 2% paraformaldehyde (w/v) in 0.1 M cacodylate buffer, pH 7.4, and then post-fixed for 2 h at room temperature (RT) in 2% (w/v) osmium tetroxide. After dehydration (in graded ethanol series), tissues were infiltrated overnight in propylene oxide (Sigma Aldrich, St. Louis, MO, USA) and TAAB embedding resin, then transferred to embedding moulds in pure TAAB embedding resin (3 h) (TAAB Laboratories Equipment Ltd., UK)] and polymerized (48 h, 60 °C). Ultrathin Sect. (70 nm) were cut with a UC 7 ultramicrotome (Leica Microsystems, Austria) and stained with platinum blue (EMS, USA) for 15 min and lead citrate (Leica Ultrostain 2) for 5 min. Electron micrographs were taken using a Tecnai G2 transmission electron microscope (Thermo Fisher Scientific, Netherlands) with a Gatan Ultrascan 1000 Charge Coupled Device (CCD) camera (−20 °C, Digital Micrograph acquisition software, Ametek Gatan, Germany and Serial EM) at an acceleration voltage of 120 kV.

### Assessment of mitochondrial respiration

Mitochondrial function and glycolysis of WT and KO lung endothelial cells were assessed using the Seahorse XF Pro instrument (Agilent, Santa Clara, CA, USA) by measuring oxygen consumption rate (OCR) and extracellular acidification rate (ECAR) respectively. Sensor cartridges were hydrated overnight with Seahorse XF calibrant at 37 °C. 20,000 mL ECs were seeded per well on 96 well seahorse plate (XFe96/XF Pro, 103794-100) one day prior and incubated at 37 °C. Cells were serum starved for 6 h before cell medium was replaced with seahorse assay media and incubated for 45 min in a non-CO_2_ incubator at 37 °C prior to measurement. Seahorse assay media for glycolysis stress test was supplemented with 2mM L-glutamine (Gibco, New York city, NY, USA) and assay media for cell mitochondrial stress was supplemented with 2mM L-glutamine, 1mM sodium pyruvate (Gibco, New York city, NY, USA) and 10mM D-glucose (Sigma Aldrich, St. Louis, MO, USA). Glycolysis stress test and cell mitochondrial stress test protocols were carried out according to manufacturer instructions using the following compounds: glycolysis stress test: 10mM D-glucose, 1µM oligomycin, 50mM 2-deoxy glucose; Cell mito stress: 1.5 µM oligomycin, 0.5µM FCCP and 0.5µM antimycin. Glucose, oligomycin, carbonyl cyanide-p-(trifluoromethoxy)phenylhydrazone (FCCP) and antimycinA were purchased from Sigma Aldrich, MO, USA and 2 Diacylglycerol from Thermo Fisher, Waltham, MA, USA. OCR and ECAR values are normalized to protein content using BCA assay (Merck, Rahway, NJ, USA) and presented as pmolO_2_/min/µg protein and mpH/min/µg protein.

### Assessment of mitochondrial membrane potential

Mice pulmonary endothelial cells were incubated with the mitochondrial membrane potential sensitive dye- TMRM (200 nM) (Invitrogen, Waltham, MA, USA) at 37 °C for 30 min. A single glass coverslip was placed on the stage of a Zeiss 200 M inverted epifluorescence microscope with a PolyChrome V monochromator light source (Till Photonics, Kaufbeuren, Germany) in a sealed, temperature-controlled RC-21B imaging chamber (Warner Instruments, Hamden, CT, USA). Fluorescence images were acquired every 3 s at excitation of 550 nm and emission of 575 nm and recorded via an air-cooled Andor Ixon camera (Andor Technology, Belfast, Ireland). Ten µM FCCP (Sigma Aldrich, St. Louis, MO, USA) was added 1 min after starting the measurement for 14 min. Cells that did not respond to FCCP with a decrease in TMRM fluorescence were excluded from further analysis. The background fluorescence of each coverslip was measured and subtracted before performing the calculations. TMRM values were calculated as mean of the fluorescence readings within the first minute and delta TMRM was calculated as the difference between the basal TMRM value and the last point measurement of FCCP treatment. Images were stored and analysed offline using TillVision software (Till Photonics, Germany).

### Assessment of ROS production

Mice pulmonary endothelial cells were incubated with the ROS sensitive dye H_2_DCFDA (1 µM) (Biotium, Fremont, CA, USA) at 37 °C for 30 min. A single coverslip was placed on the stage of a Zeiss 200 M inverted epifluorescence microscope with a PolyChrome V monochromator light source (Till Photonics, Kaufbeuren, Germany) in a sealed, temperature-controlled RC-21B imaging chamber (Warner Instruments, Hamden, CT, USA). Fluorescence images were acquired every three seconds for a total of one minute, at an excitation of 490 nm and an emission of 520 nm via an air-cooled Andor Ixon camera (Andor Technology, Belfast, Ireland). The background fluorescence of each coverslip was measured and subtracted prior to calculations. For each measured cell, the average fluorescence over one minute was calculated and analysed. Images were stored offline using TillVision software (Till Photonics, Germany).

### Statistics

Data are presented as individual data points next to the mean. Results are expressed as mean ± standard error of the mean (S.E.M.), with sample size (n) or violin plots showing individual values and medians in the respective figure legends. Statistical analyses were conducted using GraphPad Prism (version 10.2.3; GraphPad Software, La Jolla, CA). Appropriate statistical tests (Mann-Whitney U- test, two- way ANOVA with Bonferroni post-hoc test, unpaired and paired t-tests, Spearman correlation) were selected based on the data set, as noted in the figure legends. The assumptions for all tests were met and the group variances were comparable. Statistical significance was defined as *p* < 0.05, with p-values as follows: *p ≤ 0.05, **p ≤ 0.01, and ***p ≤ 0.001.

## Results

**Fig. 1:**
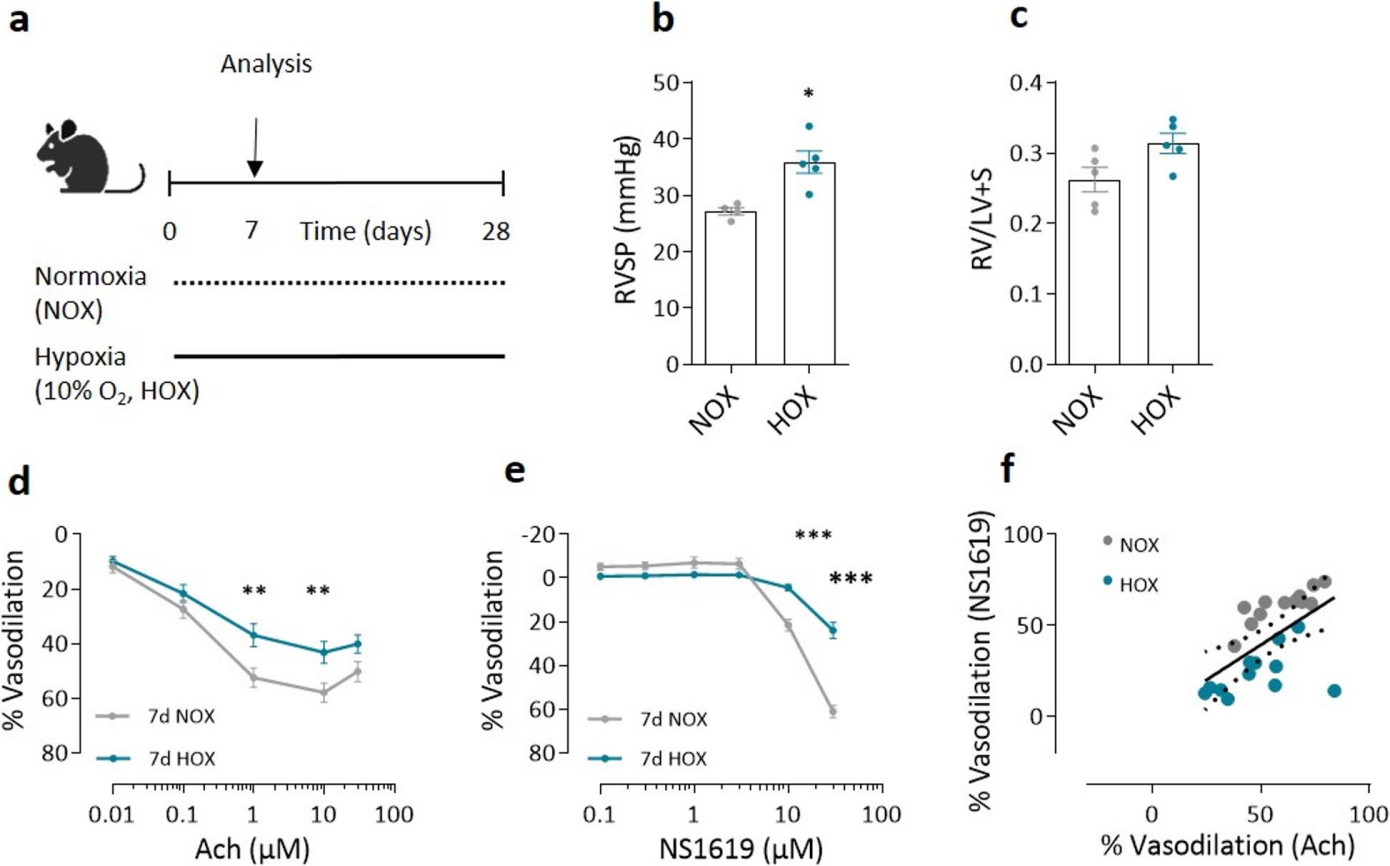
Impairment of endothelial BK function in pulmonary arteries of mice exposed to hypoxia on day 7. **a** Schematic representation of the experimental protocol. Mice were randomized into two groups. The mice in the experimental group were exposed to hypoxia (HOX) for 7 days, while the mice in the control group were kept under normoxic conditions (NOX) (*n* = 5 in each group). NOX and HOX labels refer to these conditions in the figure. On day 8, hemodynamic analyses, pulmonary artery dissection and organ harvesting were performed in all mice as indicated. **b** Assessment of right ventricular systolic pressure (RVSP) with in vivo hemodynamic analysis and **(c)** estimation of right ventricular hypertrophy (Fulton index; the weight ratio of the right ventricle (RV) to the left ventricle (LV) plus septum (S)). Blue are the results from mice kept under hypoxia and grey are the results from mice kept under normoxic conditions. **d** Effect of acetylcholine (Ach) at cumulative doses on phenylephrine (PE) (1µM) pre-constricted mouse pulmonary arteries (PA) (*n* = 19 PAs obtained from 5 NOX mice and *n* = 18 PAs from 5 HOX mice. **e)** Effect of NS1619 at cumulative doses on U-46619 (30 nM) pre-constricted mouse pulmonary arteries (*n* = 12 PAs obtained from 5 NOX mice and *n* = 12 PAs obtained from 5 HOX mice). **f** Plot of Ach-induced (10 µM) and NS1619-induced (30µM) dilatation in isolated pulmonary arteries. The black solid line shows the correlation between the data from pulmonary arteries obtained from NOX mice (grey) and from HOX mice (blue) for 7 days with *r* = 0.63, *p* = 0.001. Spearman’s correlation coefficient, *p < 0.05 ** *p* < 0.01, *** *p* < 0.001, ANOVA with Bonferroni post-hoc test, Mann- Whitney test, data are presented as mean ± SEM. c-f are ex vivo measurements

**Fig. 2:**
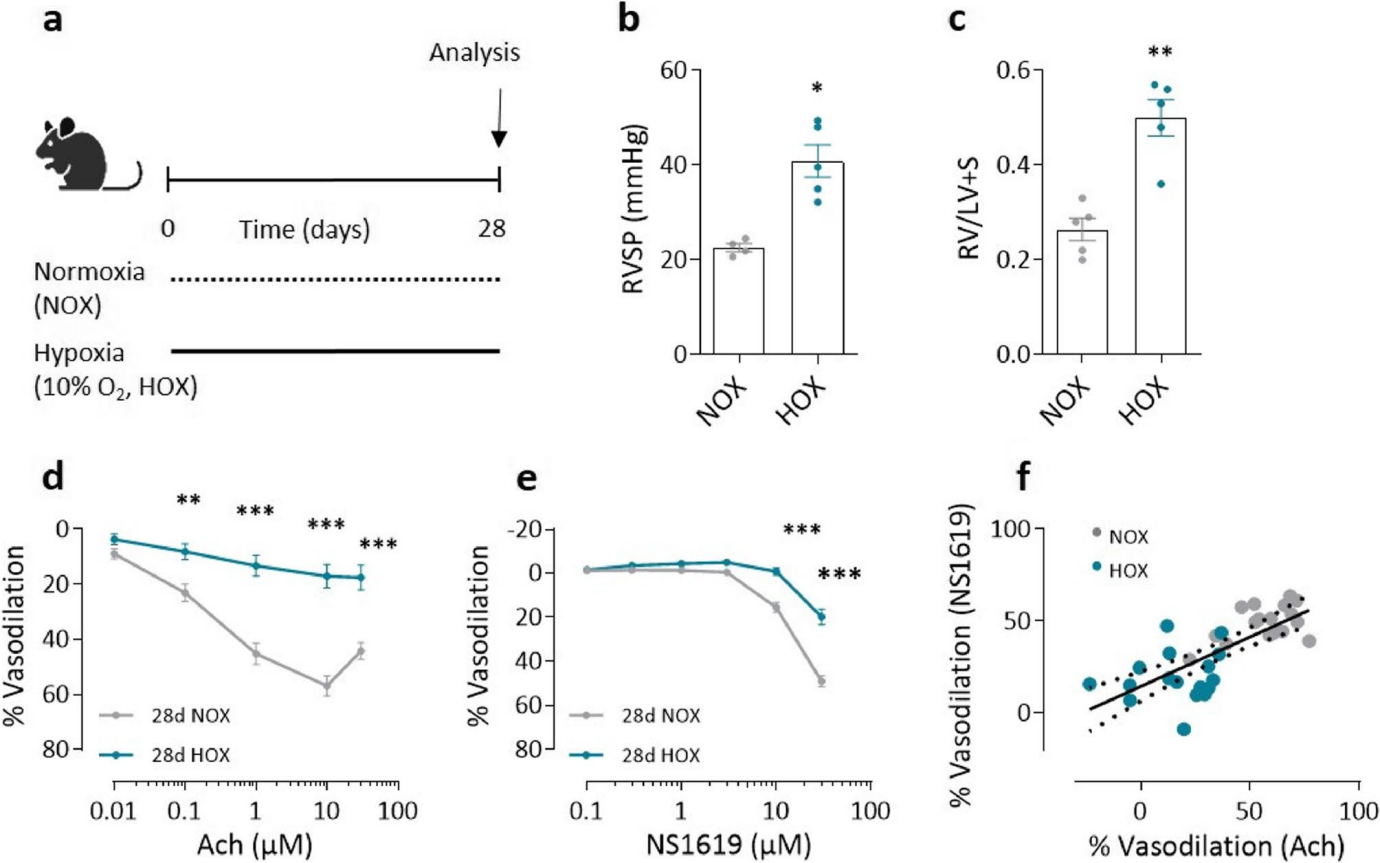
Impairment of endothelial BK function in hypoxic mouse model for pulmonary hypertension on day 28**. a** Schematic representation of the experimental protocol. Mice were randomized into two groups. The mice in the experimental group were exposed to hypoxia (HOX) for 4 weeks, while the mice in the control group were kept under normoxic conditions (NOX) (*n* = 5 in each group). NOX and HOX labels refer to these conditions in the figure. On day 29, hemodynamic analyses, pulmonary artery dissection and organ harvesting were performed in all mice as indicated. **b** Assessment of right ventricular systolic pressure (RVSP) with in vivo hemodynamic analysis and **(c)** estimation of right ventricular hypertrophy (Fulton index; the weight ratio of the right ventricle (RV) to the left ventricle (LV) plus septum (S)). Blue are the results from mice kept under hypoxia and grey are the results from mice kept under normoxic conditions. **d** Effect of acetylcholine (Ach) at cumulative doses on phenylephrine (PE) (1µM) pre-constricted mouse pulmonary arteries (*n* = 17 PAs obtained from 5 NOX mice and *n* = 17 PAs obtained from 5 HOX mice) and **(e)** Effect of NS1619 at cumulative doses on U-46,619 (30 nM) pre-constricted mouse pulmonary arteries (*n* = 17 PAs obtained from 5 NOX mice and *n* = 17 PAs obtained from 5 HOX mice). **f** Plot of Ach-induced (10 µM) and NS1619-induced (30 µM) dilatation in isolated pulmonary arteries. The black solid line shows correlation between the pulmonary arteries obtained from NOX mice (grey) and from HOX mice (blue) for 28 days with *r* = 0.75, *p* = 0.0001. Spearman’s correlation coefficient, *p < 0.05, ** *p* < 0.01, *** *p* < 0.001, ANOVA with Bonferroni post-hoc test, Mann- Whitney test, data are presented as mean ± SEM. c-f are ex vivo measurements

Mice were exposed to chronic hypoxia (HOX) for 7 and 28 days (Figs. 1a and 2a). As expected, at both time points, hypoxic mice exhibited a significant increase in right ventricular systolic pressure (RVSP) compared to normoxic controls (NOX), which was associated with increased pulmonary arterial pressure (Figs. 1b and 2b). The Fulton index, a measure of right ventricular hypertrophy, was elevated, confirming early structural changes in the right ventricle indicative of the development of PH (Figs. 1c and 2c).

### Impairment of endothelial BK function and concomitant endothelial dysfunction under hypoxia

The functional state of the pulmonary endothelium was assessed by evaluating endothelium-dependent vasodilation of 1 µM phenylephrine pre-constricted PAs (PE provoked a vasoconstrictive response of 1.8 ±_0.1 mN in PAs at day 7 of normoxia, 1.8 ± 0.1 mNat day 7 of hypoxia, 2.0 ± 0.1 mN at day 28 of normoxia and 1.7 ± 0.3 mN at day 28 of hypoxia, respectively). Mice exposed to hypoxia showed significantly impaired vasodilation as early as day 7 in response to increasing doses of Ach, compared to normoxic controls (Fig. 1d). This endothelial dysfunction persisted until day 28 (Fig. 2d), suggesting that the impairment of endothelial function in chronic hypoxia occurs early and persists with prolonged exposure. Since large-conductance calcium-activated potassium (BK) channels are known to decrease vascular tone, we administered NS1619, a BK channel activator. NS1619 induced dose-dependent vasodilation in U46619 pre-constricted PAs (U46619 provoked a vasoconstrictive response of 2.9 ± 0.2 mN in PAs at day 7 of normoxia, 3.6 ± 0.2 mN at day 7 of hypoxia, 3.1 ± 0.2 mN at day 28 of normoxia and 4.3 ± 0.3 mN at day 28 of hypoxia, respectively). This vasodilator response was significantly reduced in hypoxic mice as early as day 7 and persisted until day 28 (Figs. 1e and 2e). A positive correlation between Ach -induced vasodilation and NS1619-induced vasodilation was observed at both time points (Figs. 1f and 2f). This suggests an interplay between BK channel activation and overall endothelial function. Despite impaired BK channel-mediated vasodilation, sodium nitroprusside (SNP)-induced vasodilation, which directly measures nitric oxide (NO)-mediated smooth muscle relaxation, remained comparable in chronic hypoxic and normoxic mice at both time points (Supplementary Figure S1a and S1b). This suggests that smooth muscle responsiveness to NO is preserved under hypoxic conditions, whereas endothelial function is impaired at least from day 7 of hypoxia.

### BK channel is present in human pulmonary arterial endothelial cells (hPAECs)

**Fig. 3:**
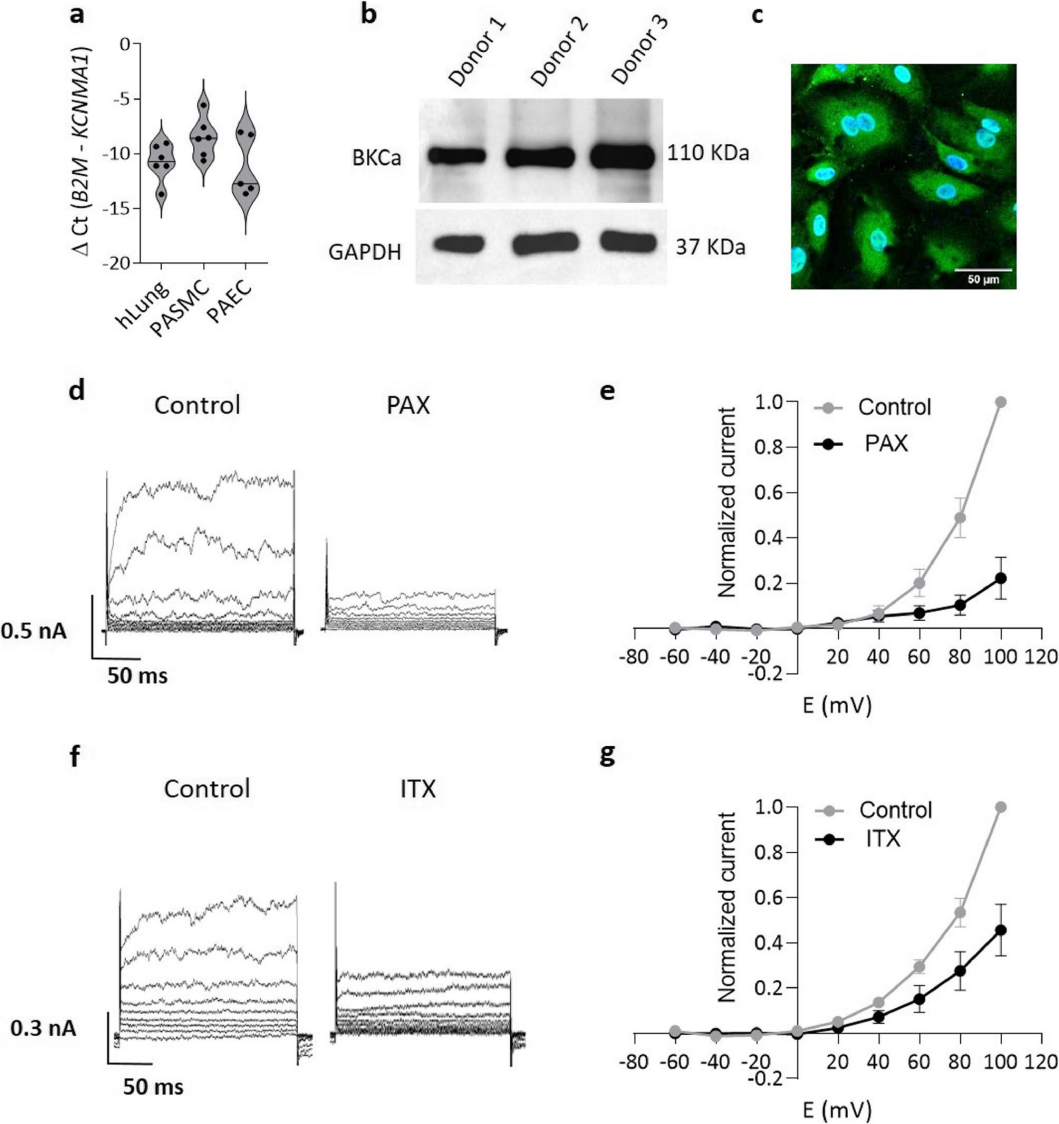
Functional BK channels in human pulmonary arterial ECs (hPAECs).** a** qPCR analysis of expression levels of *KCNMA1* gene that encodes BK channel in whole human lung homogenate, hPASMCs and in hPAECs (*n* = 5) shown as violin plots of individual values and medians. **b** Western blot analysis of expression of BK channel protein in donor hPAECs (*n* = 3). **c** Immunofluorescence staining of fixed hPAECs with anti-BK antibody and DAPI. Scale bar: 50 μm. **d** - **g** Representative whole-cell current traces from hPAECs in the absence (control) or presence of (**d**)paxilline (2 µM) or (**f**) iberiotoxin (100 nM, lower traces). The corresponding current-voltage (I-V) curves are shown (**e**,** g**) as mean +/- standard error of the mean (SEM) (*n* = 5–6 per group). Currents values were normalized to the maximal current observed in the control group

Next, we investigated the presence of BK channels in hPAECs. Gene expression analysis by qPCR demonstrated that the BK channel gene *KCNMA1* is expressed in hPAECs at levels comparable to those in human pulmonary artery smooth muscle cells (hPASMCs) and lung homogenate (Fig. 3a). Protein expression was confirmed by Western blot analysis (Fig. 3b) and immunofluorescence staining of hPAECs (Fig. 3c). Electrophysiological recordings from hPAECs revealed a dominant outward current that appeared at positive membrane potentials (with 100 nM [Ca^2+^]_i_) with no significant contribution from other ion conductance at negative membrane potentials. Application of the known BK inhibitors paxilline (PAX, 2 µM) or iberiotoxin (ITX, 100 nM), significantly reduced the total current by 77 ± 10% and 54 ± 11% at + 100 mV, respectively, indicating that BK channels were responsible for the majority of the current (Fig. 3d and f). The current-voltage (I-V) relationships further highlighted the PAX and ITX-sensitive currents (Fig. 3e and g). This suggests that the detected BK channels in hPAECs are functional.

### Reduced expression of BK channels in hPAECs of IPAH patients

**Fig. 4:**
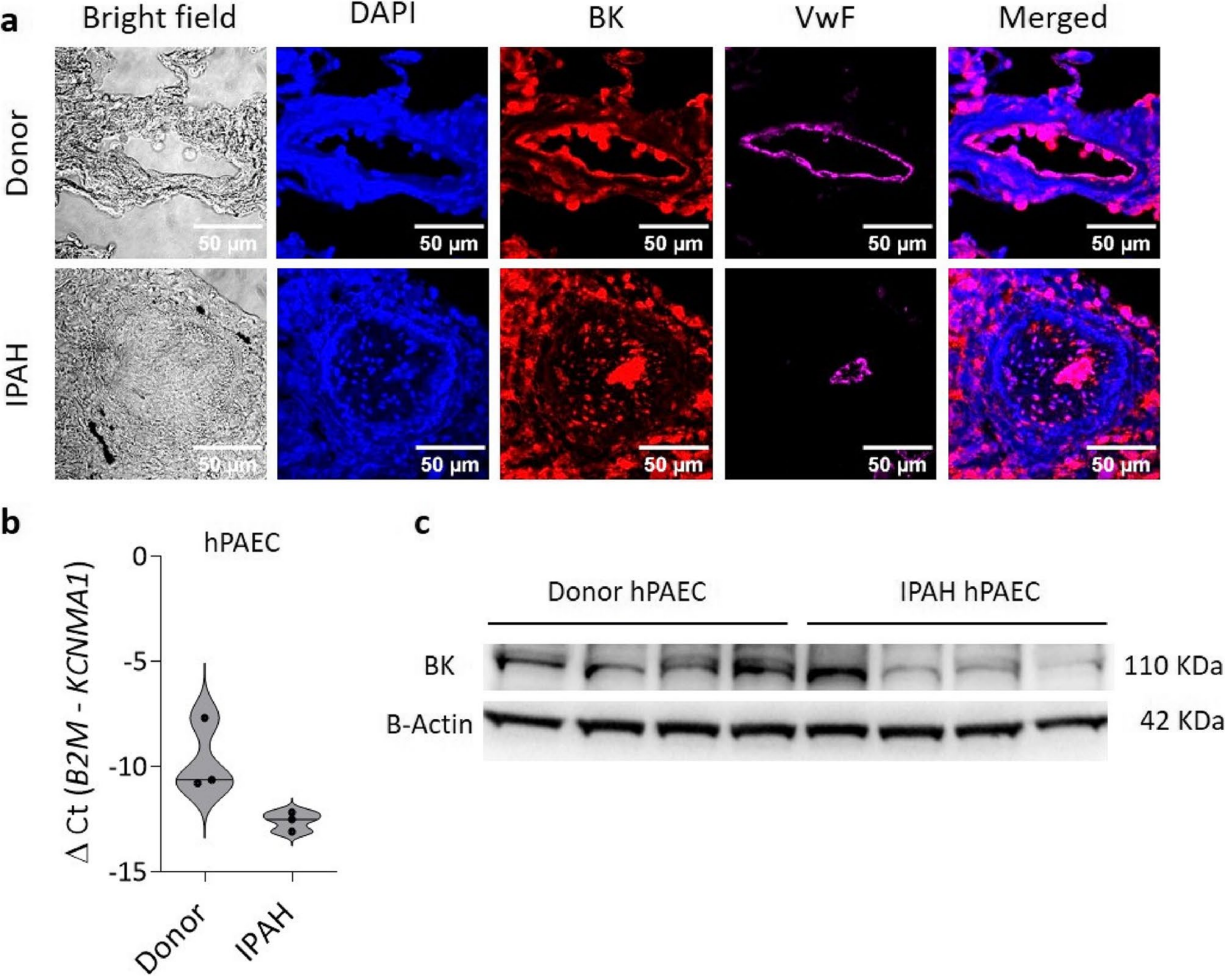
BK channel expression in IPAH hPAECs. **a** Immunofluorescence staining of donor and IPAH lung sections. Scale bar: 50 μm. **b** qPCR analysis of expression levels of *KCNMA1* gene that encodes BK channel in donor and IPAH hPAECs (*n* = 3) shown as violin plots of individual values and medians. **c** Western blot analysis of expression of BK channel protein in donor and IPAH hPAECs (*n* = 4)

Immunofluorescence stainings were performed on paraffin embedded donor and IPAH lung sections containing PAs. BK channel was found to be expressed in PA endothelium of both donor and IPAH lungs (Fig. 4a) (negative control is shown in Supplementary Figure S2). To investigate the possible role of BK channels in endothelial dysfunction, their expression was analyzed in hPAECs derived from patients with IPAH. First, we detected reduced mRNA expression of *KCNMA1* in hPAECs from IPAH patients of the Graz cohort, compared to controls (Fig. 4b). Next, we performed protein detection on independent samples obtained from the Pulmonary Hypertension Breakthrough Initiative at Stanford (Fig. 4c). Our finding suggests that BK channels are downregulated in hPAECs from IPAH patients, possibly contributing to endothelial dysfunction. Unfortunately, the availability of these cells was severely limited. To overcome this limitation and further investigate the role of BK channels in lung endothelium, we have used pharmacological agents and genetically modified animal models.

### Mice lacking BK channels develop pulmonary endothelial dysfunction

**Fig. 5:**
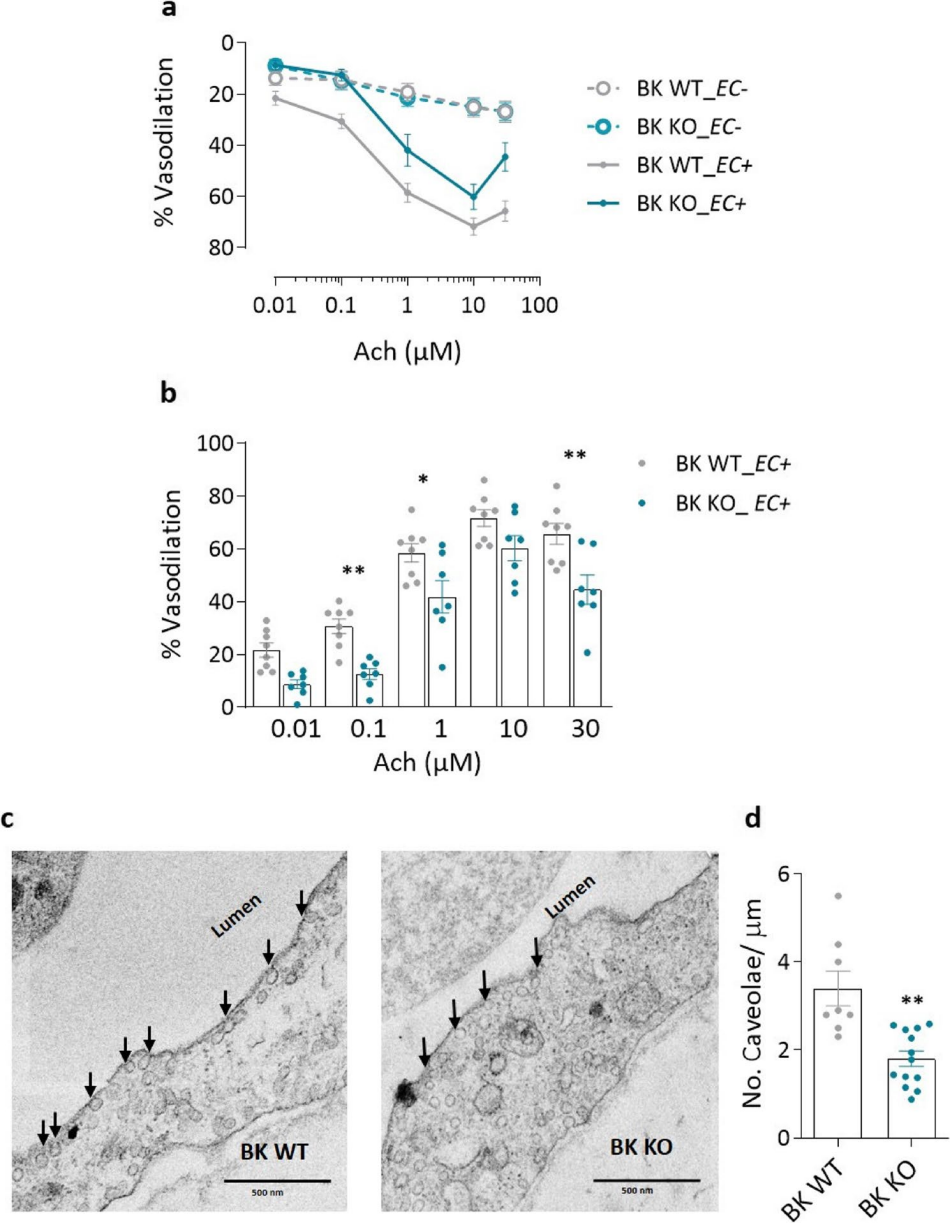
Lack of BK channel leads to attenuated vasodilation of mouse pulmonary arteries.** a** Effect of acetylcholine (Ach) in cumulative doses on phenylephrine (1 µM) pre-constricted mouse pulmonary arteries with intact endothelium (*n* = 8 PAs from 2 WT mice and *n* = 7 PAs from 2 KO mice) and with denuded endothelium (*n* = 11 PAs from 3 WT mice and *n* = 15 PAs from 4 KO mice) (**b**) The bar graph shows the difference of Ach-induced vasodilation between BK WT and BK KO PAs with intact endothelium at increasing concentrations. **c** Ultrastructural analysis of BK wild-type (WT) and BK-KO PAs by electron microscopy. Scale bar: 500 nm. **d** Endothelial surface caveolae (black arrows) were counted as omega-shaped membrane invaginations open at the luminal surface and normalized per micrometer (*n* = 24 WT PAECs and *n* = 36 KO PAECs). Mann-Whitney U-test. * *p* < 0.05, ** *p* < 0.01, ANOVA with Bonferroni post-hoc test, data are presented as mean ± SEM

To investigate whether the absence of BK channels directly causes endothelial dysfunction, BK knockout (BK KO) mice were investigated. The genetically modified model was generated as previously described [28], and the PAs of these mice were analyzed for structural and functional indicators of endothelial dysfunction (Fig. 5a). Functional assessment of PAs using isometric tension measurements showed that Ach-induced vasodilation was significantly impaired in phenylephrine pre-constricted PAs with intact endothelium from BK KO mice, compared to WT mice (Fig. 5b). PAs from BK WT and BK KO mice whose endothelium was denuded, were used as experimental controls. Vasodilation in response to sodium nitroprusside (SNP) remained unchanged among the four groups after the arteries were washed and pre-constricted with 1 µM phenylephrine (Supplementary Figure S3). To assess potential compensatory mechanisms, we next examined the expression of BK channel subunits in BK KO versus WT mice. While all β and γ subunit genes were expressed in pulmonary ECs, only the β2 and γ4 subunit genes were upregulated in BK KO mice (Supplementary Figure S4). Notably, the β2 subunit is known to inactivate BK channels [29] and the γ4 subunit exerts only a minimal effect on channel open probability [30, 31]. Thus, it is unlikely that the observed upregulation of these subunits in BK KO mice functionally compensates for the absence of the pore-forming α subunit.

Ultrastructural analysis using electron microscopy revealed a significant reduction in endothelial surface caveolae - membrane invaginations, critical for the housing of proteins such as endothelial nitric oxide synthase (eNOS) and various ion channels — in the PAs of BK KO mice compared to WT controls (Fig. 5c and d). To determine whether this reduction was due to altered gene expression of caveolae- forming proteins, we analyzed the transcript levels of *caveolin1*,* caveolin2* and *caveolin3* in pulmonary endothelial cells. No significant differences were observed between BK WT and BK KO mice (Supplementary Figure S5a). Similarly, caveolin-1 protein (important protein of caveolae) levels remained unchanged between the two groups (Supplementary Figure S5b).

### Dysfunctional mitochondria and increased ROS in lung endothelial cells of mice lacking BK channels

**Fig. 6:**
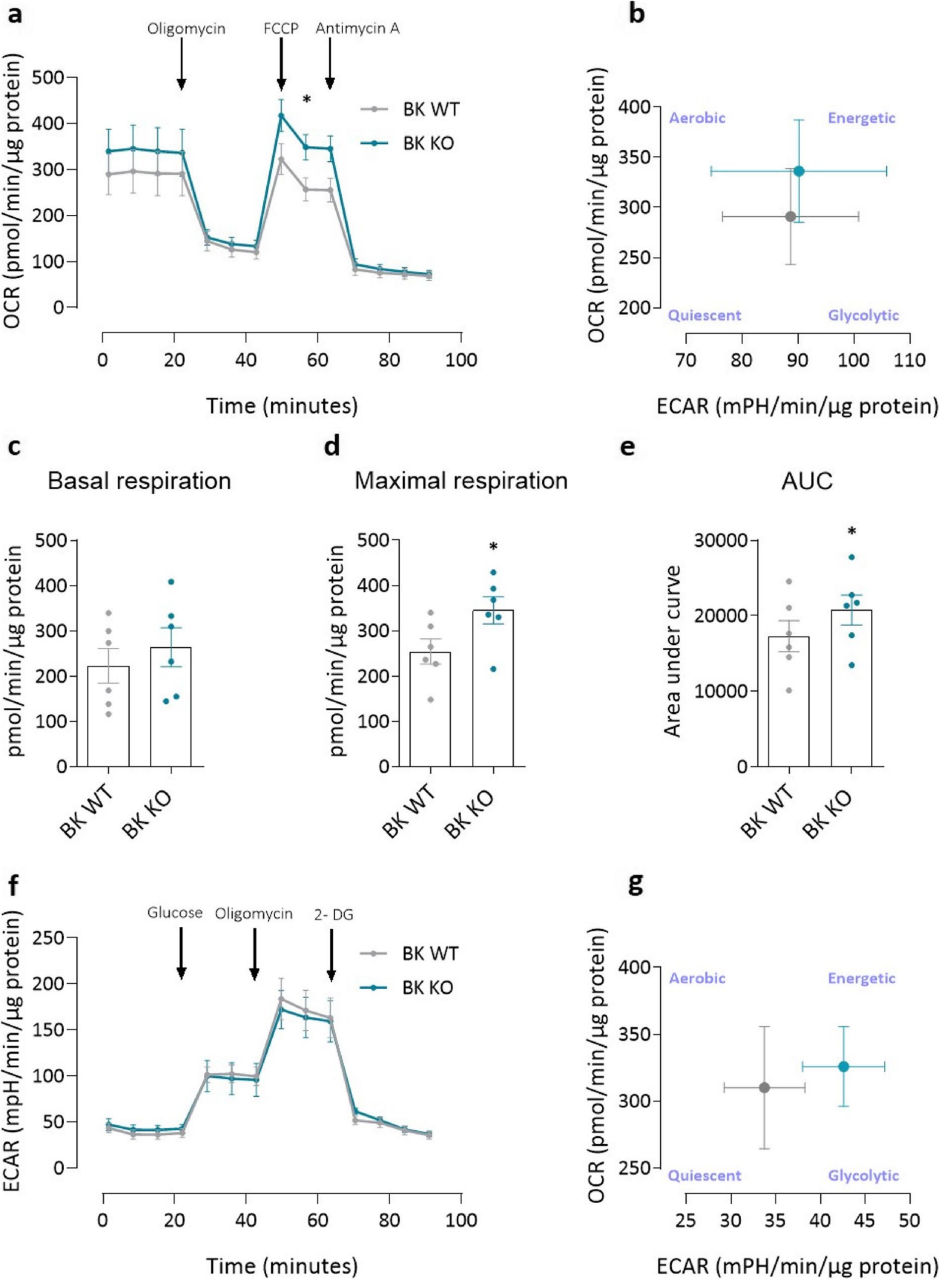
The absence of BK channel changes the metabolic function of lung endothelial cells in mice.** (a)** Mitochondrial respiration of BK WT and BK KO ECs, represented by OCR curve. (*n* = 6). **b** Energy map (ECAR/OCAR plot) under basal mitochondrial stress conditions (in the presence of glucose, pyruvate and glutamine in seahorse medium). **c- e** Bar graphs of bioenergetics parameters calculated from OCR curve between BK WT and BK KO ECs such as **(c)** basal respiration **(d)** maximal respiration and (**e**) area under the curve. **f** Glycolytic activity of BK WT and BK KO ECs represented by the ECAR curve (*n* = 6). **g** Energy map (ECAR/OCAR diagram) under basal glycolytic stress conditions (in the presence of glutamine but in the absence of glucose and pyruvate in seahorse medium). * *p* < 0.05, unpaired t- test, paired t-test, data are presented as mean ± SEM

To assess the metabolic function, two key parameters of cellular metabolism were analyzed in endothelial cells of BK WT and BK KO mice: the oxygen consumption rate (OCR), reflecting mitochondrial respiration, and the extracellular acidification rate (ECAR), indicative of glycolytic activity. Under mitochondrial stress conditions (Fig. 6a), both groups showed a similar reduction in OCR after treatment with oligomycin, indicating comparable inhibition of ATP synthase. Baseline OCR values did not differ significantly between groups (Fig. 6c); however, BK KO endothelial cells showed significantly higher maximal respiration after FCCP injection (Fig. 6d). As a result, total respiratory capacity, measured as area under the OCR curve (AUC), was significantly increased in BK KO cells (Fig. 6e). To complement these observations, ECAR profiles were analyzed under both mitochondrial (Supplementary Figure S6a) and glycolytic (Fig. 6f) stress. ECAR responses were broadly similar between BK WT and BK KO cells, although BK KO cells showed a subtle trend towards increased glycolytic activity. The OCR measurements under glycolytic stress are shown in Supplementary Figure S6b. Integrated analysis of ECAR and OCR under both conditions (Fig. 6b and g) revealed distinct bioenergetic profiles: While BK WT endothelial cells showed a predominantly quiescent metabolic phenotype, BK KO cells developed at least a tendency towards increased oxygen consumption (Fig. 6b) and glycolytic activity (Fig. 6g).

**Fig. 7:**
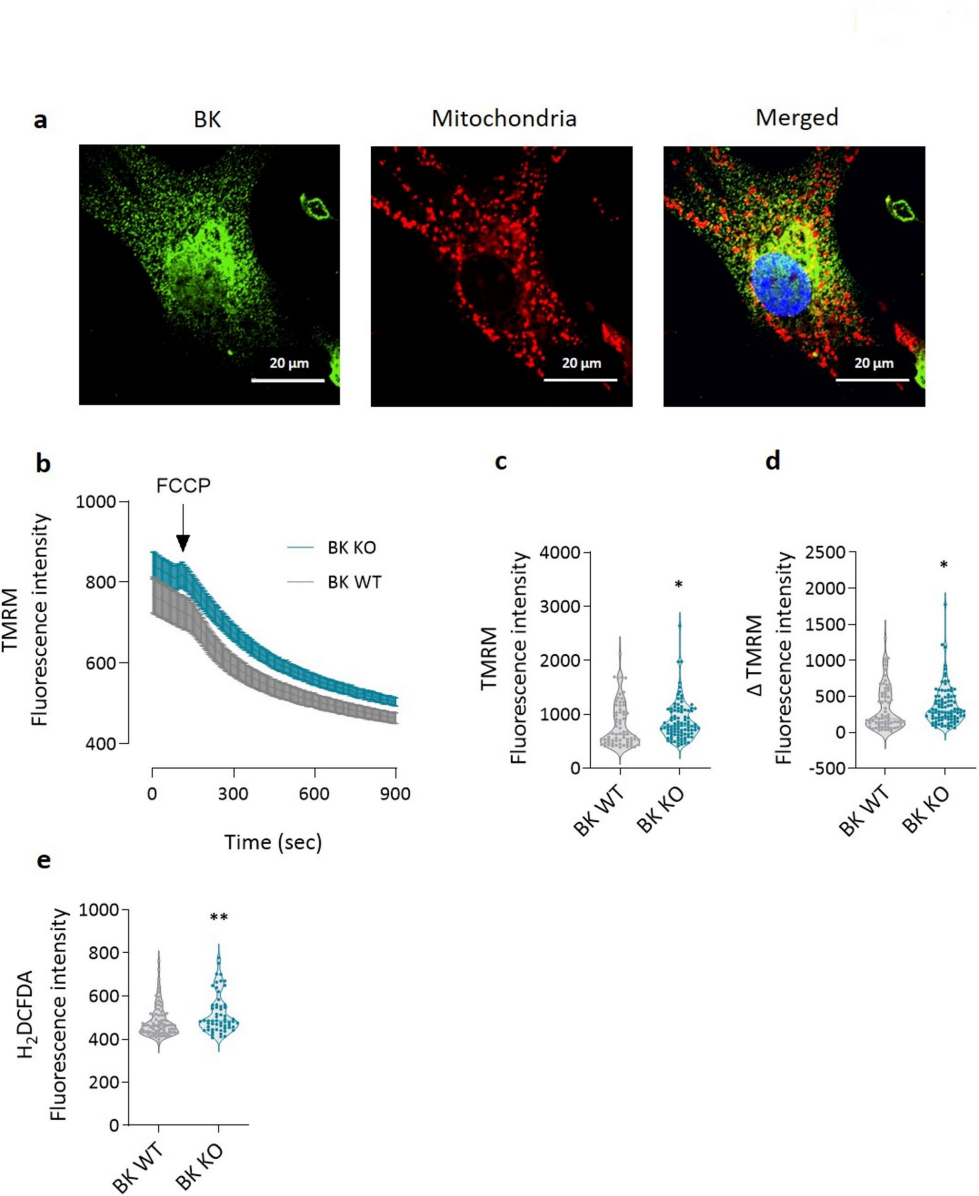
Effect of BK on endothelial mitochondrial membrane potential and ROS production.** a** Immunofluorescence staining of fixed endothelial cells with anti-BK antibody in presence of the mitochondrial marker MitoTracker Red CMXRos. Scale bar: 20 μm. **b** Mitochondrial membrane potential as determined by live cell imaging in BK WT and BK KO lung endothelial cells (*n* = 82 BK WT and *n* = 59 BK KO). Data represented as mean ± SEM. **c** Mitochondrial membrane potential at baseline. **d** Difference between basal mitochondrial membrane potential and mitochondrial membrane potential after FCCP treatment. **e** Basal ROS production by BK WT and BK KO lung endothelial cells as assessed by live cell imaging of H2DCFDA fluorescence (*n* = 69 BK WT and *n* = 98 BK KO). * *p* < 0.05, ** *p* < 0.01, Mann-Whitney U- test, data are presented as violin plots showing individual values and medians

We next investigated the expression of BK channel in mitochondria of endothelial cells. Immunofluorescence staining of ECs revealed co-localization of BK channels with the mitochondrial marker MitoTracker Red CMXRos (Fig. 7a) suggesting their mitochondrial expression. We next proceeded to study the effect of absence of BK channel on mitochondrial membrane potential in endothelial cells. Cell from BK KO animals showed higher basal TMRM fluorescence indicating mitochondrial membrane hyperpolarization compared to BK WT cells (Fig. 7b and c). Upon FCCP treatment, cells obtained from BK KO mice showed a more pronounced change in mitochondrial membrane potential compared to that observed in BK WT cells (Fig. 7d and d), a finding that is in line with the previously noticed increase in maximal respiration in the presence of FCCP. As BK KO cells thus exhibited a change in mitochondrial metabolism, we next investigated the levels of reactive oxygen species (ROS). Live cell imaging with H_2_DCFDA showed that BK KO endothelial cells produced higher ROS levels compared to BK WT cells (Fig. 7e).

### BK channels influence angiogenesis and NO production in endothelial cells

**Fig. 8:**
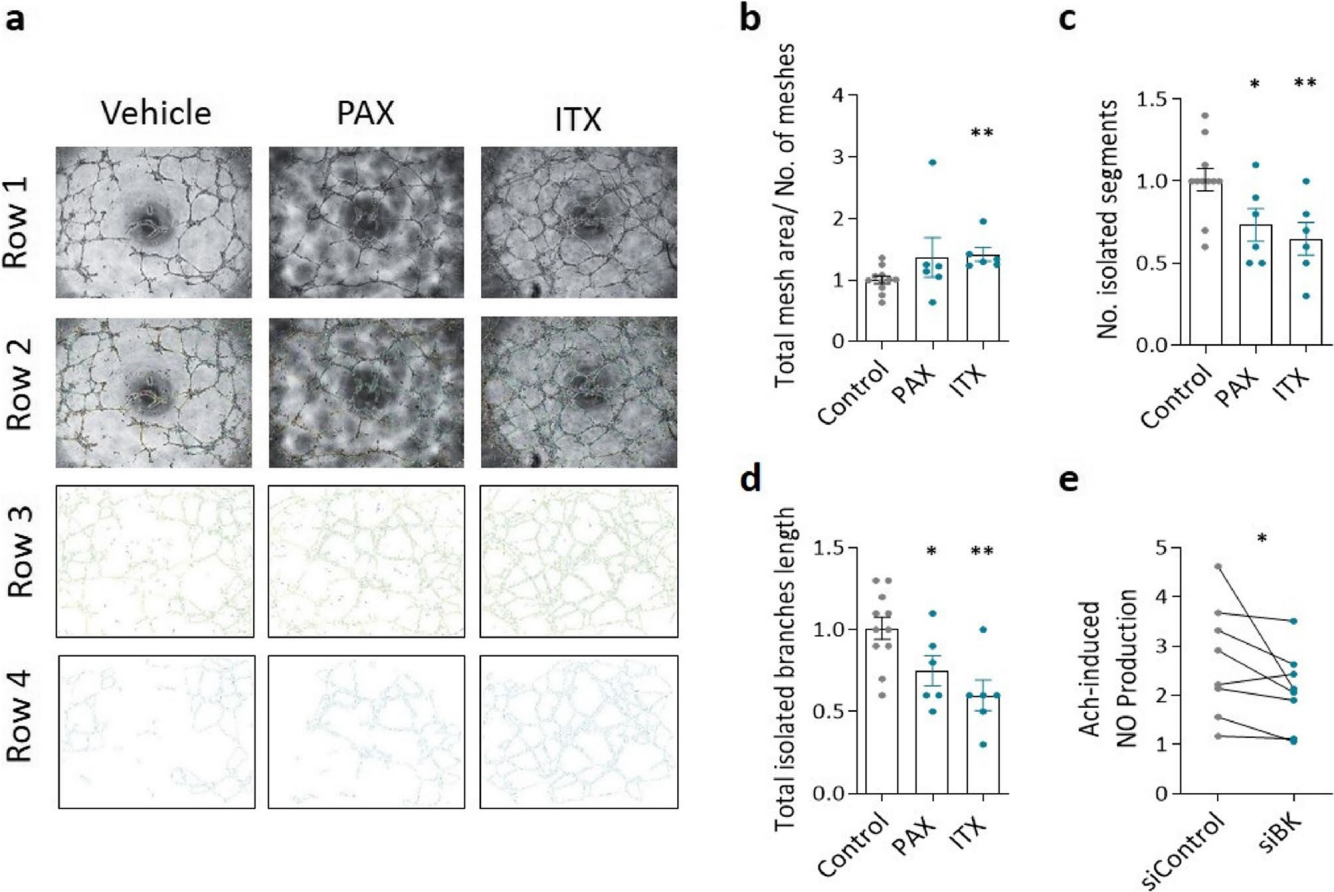
Effects of BK inhibition on hPAECs function.** a** Representative images of in vitro tubulogenesis. Row 1- Light micrographs, row 2- analyzed Light micrographs row 3- skeleton of analysis and row 4- meshes indicating closed tubes. **b-d** Analysis of the corresponding network parameters **(b)** total mesh area divided by the number of meshes, **(c)** number of isolated segments and **(d)** total isolated branch length in control and after treatment with paxilline (100 nM) or ITX (100 nM). **e** Quantitative analysis of acetylcholine (Ach)-induced NO secretion in response to BK channel silencing on Ach-induced NO production. * *p* < 0.05, ** *p* < 0.01, Mann Whitney test, paired ratio t-test, data are presented as mean ± SEM

In angiogenesis assays, the inhibition of BK channels in donor hPAECs using PAX or ITX resulted in higher mesh area, fewer isolated segments and shorter isolated branches (Figs. 8a–d). This suggests that there is no greater angiogenic potential of hPAEC under inhibition of BK channels, but a less orderly tube formation, compared with control hPAEC. Silencing BK channel expression (Supplementary Figure S7) significantly reduced NO production in response to Ach (Fig. 8e), suggesting that BK channels are essential for the Ach-induced NO production in endothelial cells. In summary, these findings underscore the important role of BK channels in maintaining endothelial function, particularly through their effects on angiogenesis and NO production.

### Lack of BK channels disrupts Piezo-1-induced [Ca^2+^]_i_ increase in ECs

**Fig. 9:**
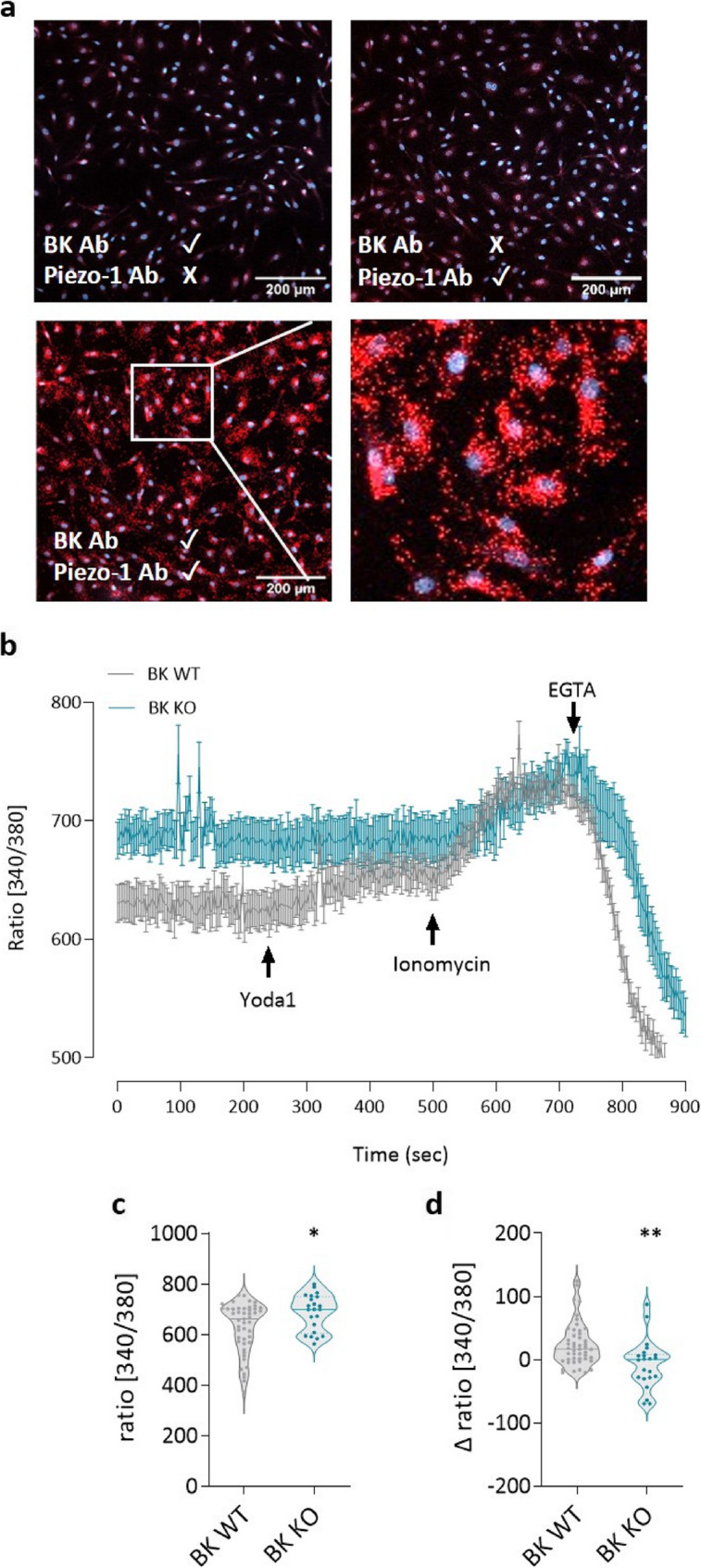
Functional link between BK and Piezo-1 channels in PA endothelium.** a** Fluorescence image of positive proximity ligation assay (PLA) signal for BK and Piezo-1 channels. Scale bar: 200 μm. **b** Live cell calcium imaging curve of BK WT and BK KO lung endothelial cells (*n* = 47 BK WT and *n* = 22 BK KO). **c** Calcium signal at baseline. **d** Calcium signal upon treatment with 10µM Yoda1 (Piezo-1 activator). * *p* < 0.05, ** *p* < 0.01, unpaired Student t-test, data are presented as means ± SEM and as violin plots showing individual values and medians

Using the proximity ligation assay (PLA), we confirmed the co-localization of BK and Piezo-1 channels in hPAECs, as evidenced by a strong fluorescent proximity ligation signal (Fig. 9a). Therefore, we hypothesized that the two channels might be functionally linked and performed live cell calcium imaging on ECs from BK WT and BK KO mice (Fig. 9b). Lung ECs from BK KO mice had higher basal calcium levels compared to those from BK WT mice (Fig. 9c). No differences in mRNA expression of calcium influx channels such as *Piezo-1*, *Piezo-2*, *STIM1* or *ORAI1* were observed between BK KO vs. BK WT mouse lung homogenates (Supplementary Figure. S8a) or in lung endothelial cells (including *TRPV4*) (Supplementary Figure S8b). After treatment with the Piezo-1 specific activator Yoda 1 (10 µM), an increase in [Ca^2+^]_i_ was observed in ECs from BK WT animals compared to BK KO mice (Fig. 9d). Piezo-1 protein expression remained the same between BK WT and BK KO lung endothelial cells (Supplementary Figure S8c). This suggests a functional role of BK channels for the Piezo-1 function in endothelial calcium signaling.

## Discussion

This study provides comprehensive evidence that large-conductance calcium-activated potassium (BK) channels are essential regulators of pulmonary endothelial cell function and contribute significantly to the pathology of pulmonary hypertension (PH). The main findings are the following: (1) Functional BK channels are expressed in human pulmonary artery endothelial cells (hPAECs) and are downregulated in hPAECs from patients with idiopathic pulmonary arterial hypertension (IPAH). (2) Endothelial dysfunction and impaired BK channel-mediated vasodilation are observed in PAs from mice exposed to hypoxia for 7 and 28 days. (3) Pharmacological inhibition or siRNA-mediated silencing of BK channels in hPAECs impairs acetylcholine-induced nitric oxide (NO) production and leads to impaired angiogenesis. (4) BK KO mice exhibit impaired endothelium-dependent vasodilation, reduced density of endothelial caveolae and elevated basal intracellular calcium concentrations [Ca^2+^]_i_. (5) ECs from BK KO mice show a metabolic shift towards increased mitochondrial respiration and glycolytic activity. (6) These cells also display mitochondrial membrane hyperpolarization and increased ROS production. (7) Finally, BK channels colocalize with Piezo-1 channels, and their absence blunts Piezo-1-mediated calcium influx.

We observed significant endothelial dysfunction in the PAs of mice exposed to hypoxia, detectable as early as day 7, evidenced by an attenuated Ach response, while SNP-mediated vasodilation remained intact. Importantly, vasodilation, induced by the BK channel activator NS1619, was also markedly reduced under hypoxic conditions. These early alterations in BK function may contribute to progressive vascular remodeling and PH during prolonged hypoxia and in IPAH patients. A previous study support this view: overexpression of miR-29b, elevated in IPAH-PASMCs, downregulated the BK channel β1 subunit expression and current density in donor PAMSCs [32]. In contrast, transcriptomic profiling of PAH lungs showed increased BK mRNA levels [33], although this likely reflects expression in non-endothelial cells, including immune or structural cell types. Our results are more consistent with studies using the monocrotaline (MCT)-induced PH rat model, where targeted activation of BK channels attenuated the development of PH [34–36]. Our results suggest that these protective effects may be mediated, at least in part, by endothelial and not smooth muscle BK signaling.

We also resolve a long-standing debate over whether BK channels are expressed in human PAECs [22, 23, 37, 38]. Using qPCR, Western blotting, immunofluorescence and patch-clamp recordings, we show BK channel expression and functionality in human PAECs. Moreover, we show downregulation of the BK channel at both gene and protein levels in IPAH-derived hPAECs, which likely contributes to the impaired endothelial function observed in these patients.

Our metabolic analyses reveal that BK KO endothelial cells are characterized by elevated FCCP-induced oxygen consumption, suggesting a shift toward increased mitochondrial respiration. This aligns with a broader bioenergetic reprogramming, which may elevate energy demand as observed in previous studies using PH models [39, 40]. While mitochondrial BK channels are known to be present in other ECs [41–44], we confirm the mitochondrial localization of BK in pulmonary ECs and show that their absence results in mitochondrial hyperpolarization, greater susceptibility to depolarization by FCCP treatment and increased ROS production [45, 46] – all consistent with mitochondrial dysfunction. Whether these effects stem directly from loss of mitochondrial BK channels or from global alterations in cellular homeostasis remains unclear at this stage and warrants further investigation.

We observed a significant reduction in endothelial caveolae in BK KO pulmonary arteries, a structural pathology not previously reported. Transcript and protein levels of caveolin-1 were unchanged, suggesting that the reduction in caveolae density was not due to altered caveolin expression. Instead, it could be due to non-transcriptional mechanisms, such as impaired caveolin transport, altered membrane lipid composition, or increased turnover. This suggests that BK channels are structural determinants of caveolar integrity and essential for eNOS signaling. Similar results were reported in KCNK3/TASK-1 knockout endothelial cells, in which the density of caveolae was reduced despite preserved caveolin expression [47], arguing for a broader role of potassium channels in regulating membrane microdomain architecture.

It is well established that pulmonary hypertension is strongly associated with endothelial dysfunction [9–13, 48, 49]. In this study we show, that BK channels are strongly involved in this pathology. Absence of BK channels impairs pulmonary endothelial function, as shown by reduced acetylcholine-induced vasorelaxation in pulmonary arteries from BK-KO mice compared with WT controls. Consistent with this, knockdown of BK in hPAECs significantly impairs Ach-induced nitric oxide (NO) production, mimicking the reduced NO production observed in IPAH-derived PAECs [50–52]. Furthermore, inhibition of the BK channel disrupted angiogenesis in vitro, leading to disorganized vessel formation — a possible mechanism contributing to the abnormal vascular remodeling characteristic of IPAH [53, 54].

Mechanistically, we demonstrate that BK channels are colocalized with Piezo-1 channels in ECs and are required for effective Piezo-1-mediated Ca^2+^ influx. This interaction may be particularly relevant in IPAH, where Piezo-1 expression is upregulated [55]. BK channels may act to stabilize Piezo-1-dependent signaling domains, contributing to shear-induced endothelial responses, including nitric oxide (NO) production and regulation of transcription factors that respond to blood flow. Our study extends prior observations from other cell types, where Ca^2+^-activated K^+^ channels and Piezo-1 exhibit cooperative activity [56–59].

Our study has a few Limitations. Since the PAECs were obtained from end-stage IPAH patients, our findings may not reflect early disease processes. However, the observed BK dysfunction in our hypoxia-exposed mice as early as day 7 – a phase considered early in pulmonary vascular remodeling supports involvement of this mechanism, early on. Although we used primary cultured endothelial cells, they directly isolated from patients at the time of lung transplantation, providing translationally meaningful insight.

## Conclusion

**Fig. 10:**
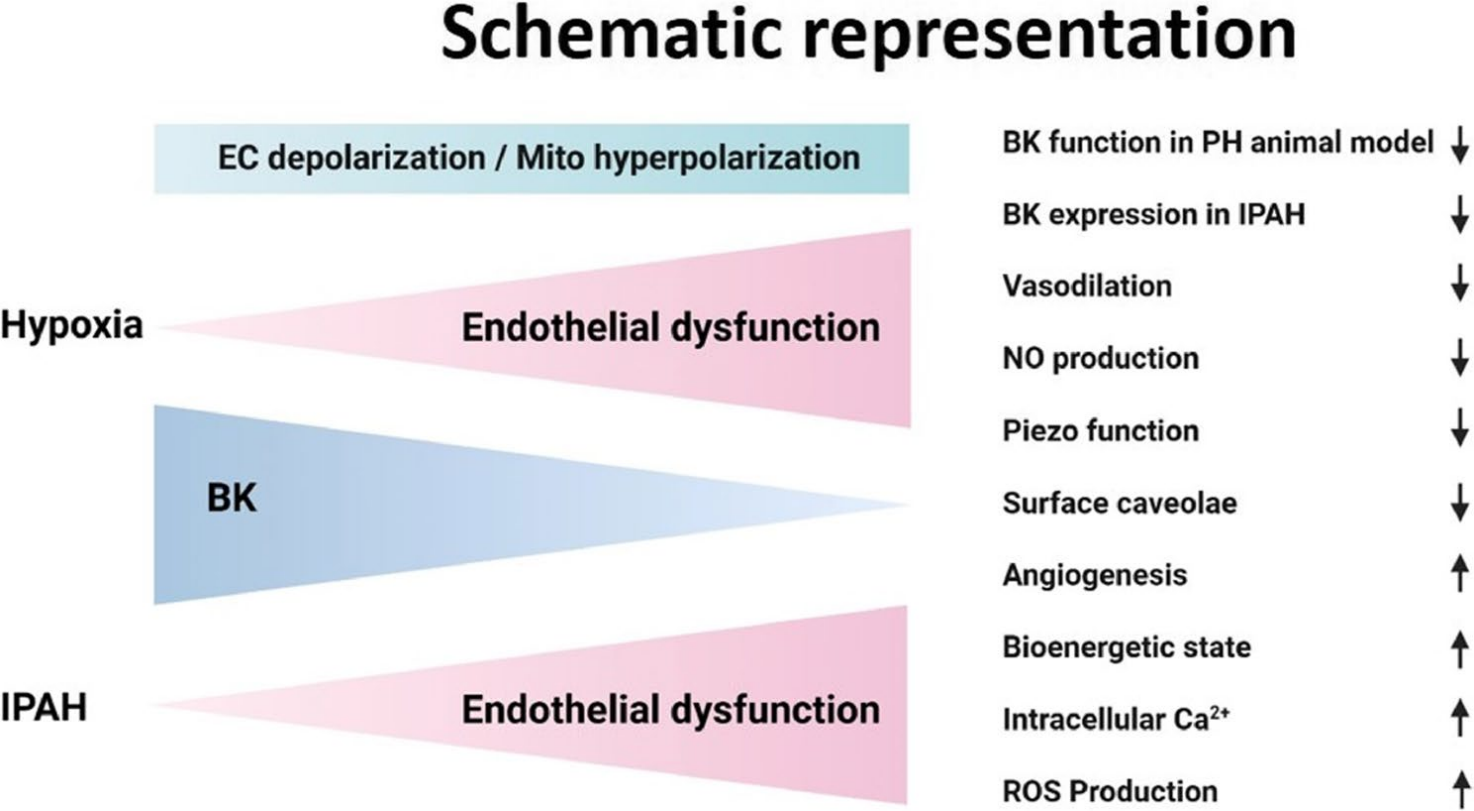
Schematic representation summarizing the findings of the present study. Arrows indicate increase or reduction

Our findings identify BK channels as critical regulators of pulmonary endothelial function and highlight their role in key pathological features of PH. Loss or downregulation of BK channels led to reduced nitric oxide production, disorganized angiogenesis, mitochondrial hyperpolarization, increased oxidative stress and a metabolic shift towards elevated respiration and glycolysis. These changes were accompanied by endothelial dysfunction both in vivo and in vitro (Fig. 10). While BK channels also modulate mechanosensitive calcium influx via Piezo-1, our findings highlight their broader role in maintaining endothelial homeostasis. Together, these results support BK channels as a potential therapeutic target to preserve vascular integrity and limit vascular remodeling in PH.

### Perspectives

BK channels appear as critical regulators of pulmonary endothelial function and vascular homeostasis, positioning them as promising therapeutic targets in pulmonary hypertension (PH). Their downregulation in idiopathic pulmonary arterial hypertension (IPAH) is linked to key pathological features of the disease. Restoring endothelial BK activity may help preserve vascular integrity and slow or reverse disease progression. The development of selective lung-targeted BK channel modulators and reliable biomarkers to guide and monitor therapy are critical for clinical translation. Targeting BK channels may offer a rationale and mechanistically sound approach to improve endothelial function in pulmonary hypertension.

## Supplementary Information

Additional file 1. Figure S1 Sodium nitroprusside (SNP) response of pulmonary arteries from C57BL/6J mice kept in normoxia (NOX) or under hypoxic conditions (HOX) for 7 or 28 days. a) Effect of SNP at cumulative doses on Phenylephrine-preconstricted (1 µM) pulmonary arteries obtained from mice kept for 7 days under normoxia or hypoxia (n=16 PAs from 5 NOX mice and n=18 PAs from 5 HOX mice). b) Effect of SNP at cumulative doses on Phenylephrine-preconstricted (1µM) pulmonary arteries obtained from mice kept for 28 days under normoxia or hypoxia (n=16 PAs from 5 NOX mice and n=13 PAs from 5 HOX mice). Data are presented as mean ± SEM. Figure S2 BK staining of human donor PAs. Negative control for BK staining in human donor PAs showing the background signal in the absence of primary antibodies. Figure S3 Sodium nitroprusside (SNP) response of pulmonary arteries obtained from BK WT and BK KO mice. Effect of SNP at cumulative doses on Phenylephrine-preconstricted (1µM) pulmonary arteries with intact endothelium (n=8 PAs obtained from 2 WT mice and n=7 PAs obtained from 2 KO mice) and with denuded endothelium (n=11 PAs obtained from 3 WT mice and n=15 PAs obtained from 4 KO mice). Data are presented as mean ± SEM. Figure S4 mRNA expression of modulatory and auxillary subunits of BK channel in lung endothelial cells obtained from BK WT and BK KO mice. qPCR showing mRNA expression levels of KCNMB1 (β1), KCNMB2 (β2), KCNMB3 (β3), KCNMB4 (β4), LRRC 26 (γ1), LRRC 52 (γ2), LRRC 55 (γ3) and LRRC 38 (γ4) in BK WT (n=4) vs BK KO (n= 6) mice lung endothelial cells. Data are represented as violin plots showing individual values and medians. Figure S5 Caveolin expression in BK WT vs BK KO endothelial cells. a) qPCR showing mRNA expression levels of CAV1, CAV2 and CAV3 in BK WT (n=4) vs BK KO (n= 6) lung endothelial cells b) Western blot analysis of protein expression of caveolin-1 in BK WT (n=4) vs BK KO (n= 4) mice lung endothelial cells. Data are represented as violin plots showing individual values and medians. Figure S6 Disrupted bioenergetics due to lack of BK. a) Glycolytic ability under mitostress condition represented by ECAR curve (n=6). b) Mitochondrial respiration under glycolytic stress condition represented by OCR curve (n=6). Data were generated on pulmonary endothelial cells obtained from BK WT and BK KO mice. Data are presented as mean ± SEM. Figure S7 Silencing of KCNMA1. qPCR of hPAECs proving the decreased expression of KCNMA1 after siRNA treatment compared to siControl in the same donor cells (n=6). ** p < 0.01 paired t-test. Figure S8 Expression of calcium influx channels in BK WT vs BK KO mice a) qPCR showing similar mRNA expression levels of piezo-1, piezo-2, STIM and ORAI channels in BK WT (n=6) vs BK KO mice (n=5) lung homogenate. b) qPCR showing mRNA expression levels of piezo-1, piezo-2, STIM1, ORAI1 and TRPV4 channels in BK WT (n=4) vs BK KO mice (n=6) lung endothelial cells. c) Western blot analysis of protein expression of piezo-1 in BK WT (n=4) vs BK KO (n= 4) lung endothelial cells. Data are represented as violin plots showing individual values and medians. Table 1: Cell and tissue used in the present study reported with corresponding gender of origin.

Additional file 2. Uncropped Western blots.

## Data Availability

No datasets were generated or analysed during the current study.

## Abbreviations

Ach: Acetylcholine
ANOVA: Analysis of variance
AUC: Area under curve
BCA: Biscinchoninic acid
BK: Large conductance calcium-activated potassium channel
Ca2+: Calcium ion
[Ca2+]i: Intracellular free calcium ion concentration
CaCl2: Calcium chloride
cDNA: Complementary deoxyribonucleic acid
COPD: Chronic obstructive pulmonary disease
DAPI: 4',6-diamidino-2-phenylindole
DMSO: Dimethyl sulphoxide
ECAR: Extracellular acidification rate
ECL: Enhanced chemiluminescence
EDTA: Ethylenediaminetetraacetic acid
eNOS: Endothelial nitric oxide synthase
FCCP: Carbonyl cyanide 4-(trifluoromethoxy)phenylhydrazone
FCS: Fetal calf serum
GAPDH: Glyceraldehyde-3-phosphate dehydrogenase
HEPES: 4-(2-hydroxyethyl)-1-piperazineethanesulfonic acid
HOX: Hypoxia
hPAEC: Human pulmonary arterial endothelial cell
IPAH: Idiopathic pulmonary arterial hypertension
ITX: Iberiotoxin
KC_a_: Calcium activated potassium channel
KCl: Potassium chloride
KCNK3: Potassium two pore domain channel subfamily member 3
KO: Knock out
MgCl2: Magnesium chloride
MOPS: 3-(N-morpholino)propanesulfonic acid, 4-Morpholino Propanesulfonic Acid
mPAP: Mean pulmonary arterial pressure
NaCl: Sodium chloride
NaOH: Sodium hydroxide
NOX: Normoxia
OCR: Oxygen consumption rate
PA: Pulmonary artery
PAEC: Pulmonary arterial endothelial cell
PAH: Pulmonary arterial hypertension
PASMC: Pulmonary arterial smooth muscle cell
PAX: Paxilline
PBS: Phosphate buffer saline
PH: Pulmonary hypertension
PLA: Proximity ligation assay
PVDF: Polyvinylidene difluoride
PVR: Pulmonary vascular resistance
qPCR: Quantitative polymerase chain reaction
RIPA: Radioimmunoprecipitation assay
RNA: Ribo nucleic acid
ROS: Reactive oxygen species
RVSP: Right ventricular systolic pressure
SDS: Sodium dodecyl sulphate
SEM: Standard error of mean
SNP: Sodium nitroprusside
TCEP: Tris(2-carboxyethyl)phosphine
WT: Wild type
WU: Wood units
2-DG: 2-Deoxy-D-glucose
