# Supplementary material for: BK channels are indispensable for endothelial function in small pulmonary arteries
Source: Cell Commun Signal. 2025 Oct 21;23:448. doi: 10.1186/s12964-025-02436-0 (PMC12542031; doi:10.1186/s12964-025-02436-0)
Supplement: Supplementary file 1 — Additional file 1. Figure S1 Sodium nitroprusside (SNP) response of pulmonary arteries from C57BL/6J mice kept in normoxia (NOX) or under hypoxic conditions (HOX) for 7 or 28 days. a) Effect of SNP at cumulative doses on Phenylephrine-preconstricted (1 µM) pulmonary arteries obtained from mice kept for 7 days under normoxia or hypoxia (n=16 PAs from 5 NOX mice and n=18 PAs from 5 HOX mice). b) Effect of SNP at cumulative doses on Phenylephrine-preconstricted (1µM) pulmonary arteries obtained from mice kept for 28 days under normoxia or hypoxia (n=16 PAs from 5 NOX mice and n=13 PAs from 5 HOX mice). Data are presented as mean ± SEM. Figure S2 BK staining of human donor PAs. Negative control for BK staining in human donor PAs showing the background signal in the absence of primary antibodies. Figure S3 Sodium nitroprusside (SNP) response of pulmonary arteries obtained from BK WT and BK KO mice. Effect of SNP at cumulative doses on Phenylephrine-preconstricted (1µM) pulmonary arteries with intact endothelium (n=8 PAs obtained from 2 WT mice and n=7 PAs obtained from 2 KO mice) and with denuded endothelium (n=11 PAs obtained from 3 WT mice and n=15 PAs obtained from 4 KO mice). Data are presented as mean ± SEM. Figure S4 mRNA expression of modulatory and auxillary subunits of BK channel in lung endothelial cells obtained from BK WT and BK KO mice. qPCR showing mRNA expression levels of KCNMB1 (β1), KCNMB2 (β2), KCNMB3 (β3), KCNMB4 (β4), LRRC 26 (γ1), LRRC 52 (γ2), LRRC 55 (γ3) and LRRC 38 (γ4) in BK WT (n=4) vs BK KO (n= 6) mice lung endothelial cells. Data are represented as violin plots showing individual values and medians. Figure S5 Caveolin expression in BK WT vs BK KO endothelial cells. a) qPCR showing mRNA expression levels of CAV1, CAV2 and CAV3 in BK WT (n=4) vs BK KO (n= 6) lung endothelial cells b) Western blot analysis of protein expression of caveolin-1 in BK WT (n=4) vs BK KO (n= 4) mice lung endothelial cells. Data are represented as violin plots showing [file 12964_2025_2436_MOESM1_ESM.docx]

**Supplementary data**

**
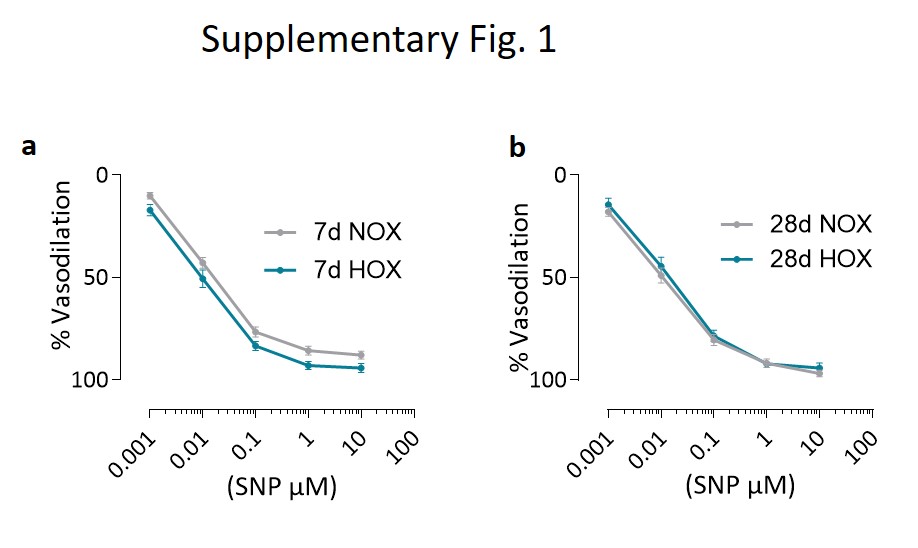
**

**Figure S1** **Sodium nitroprusside (SNP) response of pulmonary arteries from C57BL/6J mice kept in normoxia (NOX) or under hypoxic conditions (HOX) for 7 or 28 days. a)** Effect of SNP at cumulative doses on Phenylephrine-preconstricted (1 µM) pulmonary arteries obtained from mice kept for 7 days under normoxia or hypoxia (n=16 PAs from 5 NOX mice and n=18 PAs from 5 HOX mice). **b)** Effect of SNP at cumulative doses on Phenylephrine-preconstricted (1µM) pulmonary arteries obtained from mice kept for 28 days under normoxia or hypoxia (n=16 PAs from 5 NOX mice and n=13 PAs from 5 HOX mice). Data are presented as mean ± SEM.

**
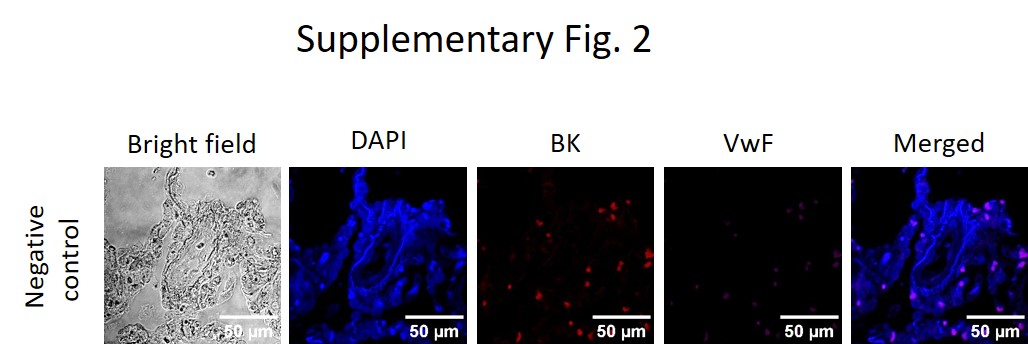
**

**Figure S2 BK staining of human donor PAs.** Negative control for BK staining in human donor PAs showing the background signal in the absence of primary antibodies.


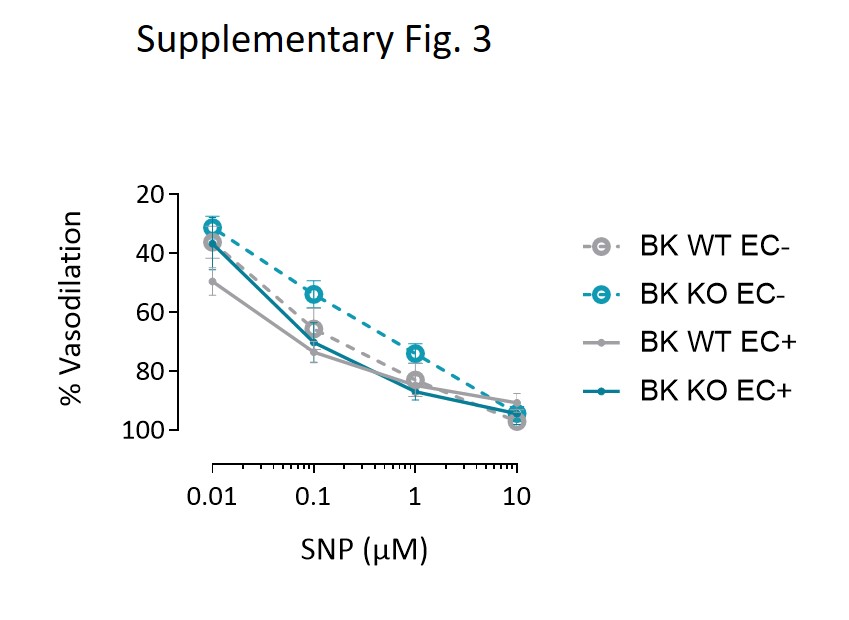


**Figure S3** **Sodium nitroprusside (SNP) response of pulmonary arteries obtained from BK WT and BK KO mice.** Effect of SNP at cumulative doses on Phenylephrine-preconstricted (1µM) pulmonary arteries with intact endothelium (n=8 PAs obtained from 2 WT mice and n=7 PAs obtained from 2 KO mice) and with denuded endothelium (n=11 PAs obtained from 3 WT mice and n=15 PAs obtained from 4 KO mice). Data are presented as mean ± SEM.


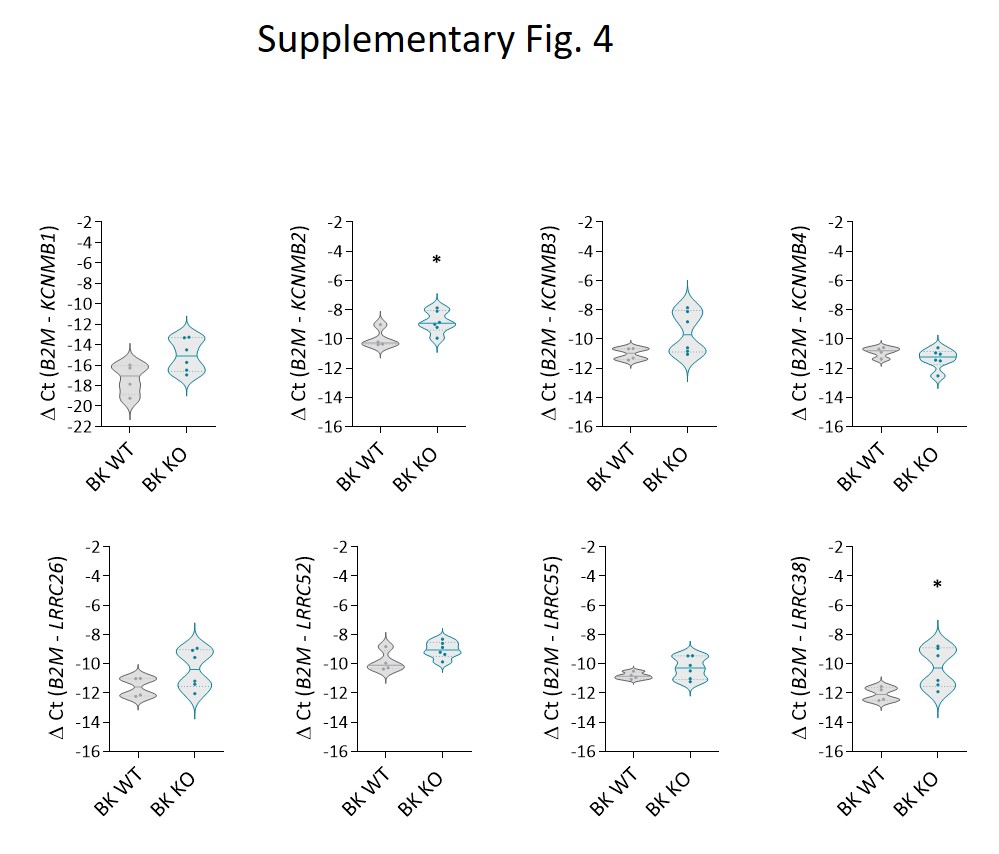


**Figure S4** **mRNA expression of modulatory and auxillary subunits of BK channel in lung endothelial cells obtained from BK WT and BK KO mice.** qPCR showing mRNA expression levels of *KCNMB1* (β1), *KCNMB2* (β2), *KCNMB3* (β3), *KCNMB4* (β4), *LRRC 26* (γ1), *LRRC 52* (γ2), *LRRC 55* (γ3) and *LRRC 38* (γ4) in BK WT (n=4) vs BK KO (n= 6) mice lung endothelial cells. Data are represented as violin plots showing individual values and medians.


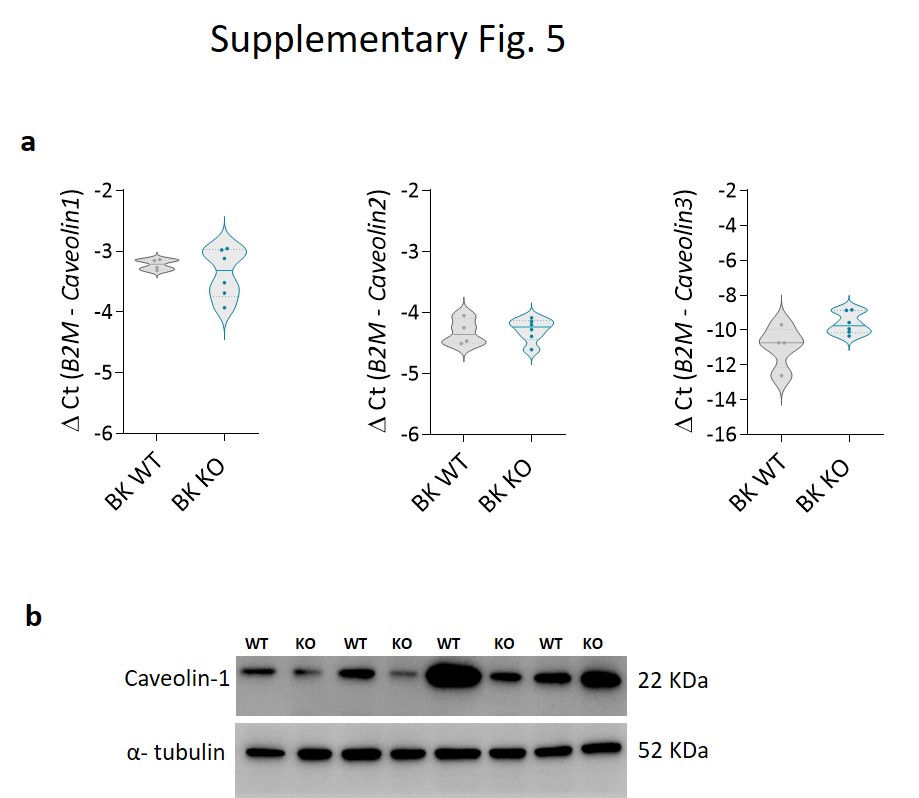


**Figure S5** **Caveolin expression in BK WT vs BK KO endothelial cells. a)** qPCR showing mRNA expression levels of *CAV1*, *CAV2* and *CAV3* in BK WT (n=4) vs BK KO (n= 6) lung endothelial cells **b)** Western blot analysis of protein expression of caveolin-1 in BK WT (n=4) vs BK KO (n= 4) mice lung endothelial cells. Data are represented as violin plots showing individual values and medians.


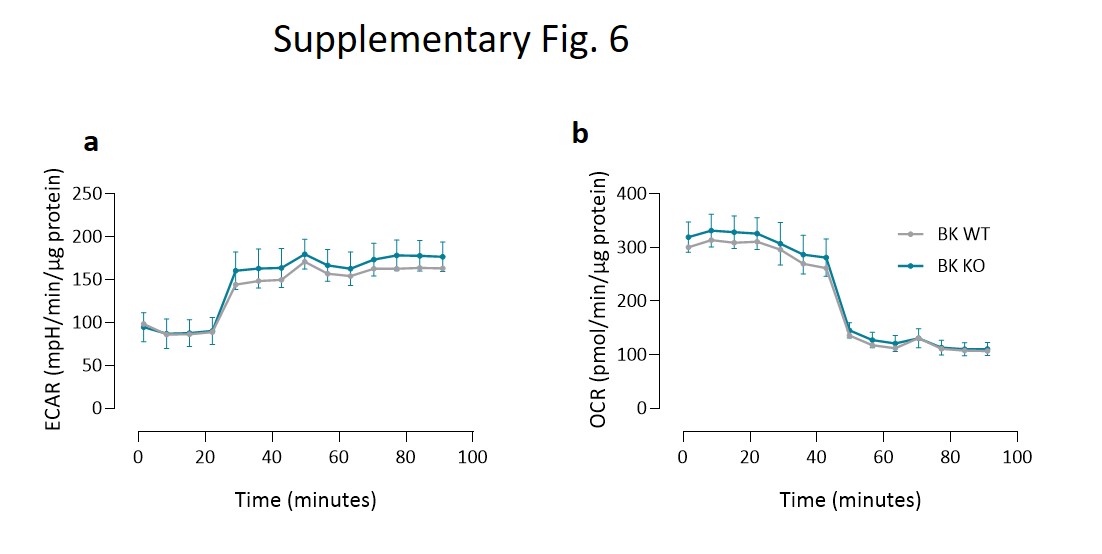


**Figure S6** **Disrupted bioenergetics due to lack of BK. a)** Glycolytic ability under mitostress condition represented by ECAR curve (n=6). **b)** Mitochondrial respiration under glycolytic stress condition represented by OCR curve (n=6). Data were generated on pulmonary endothelial cells obtained from BK WT and BK KO mice. Data are presented as mean ± SEM.


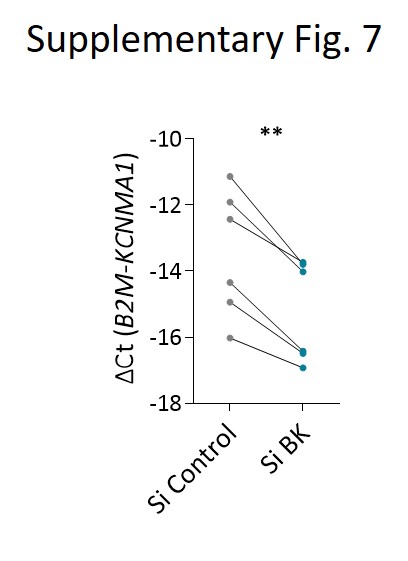


**Figure S7** **Silencing of *KCNMA1.*** qPCR of hPAECs proving the decreased expression of *KCNMA1* after siRNA treatment compared to siControl in the same donor cells (n=6). ** p < 0.01 paired t-test.


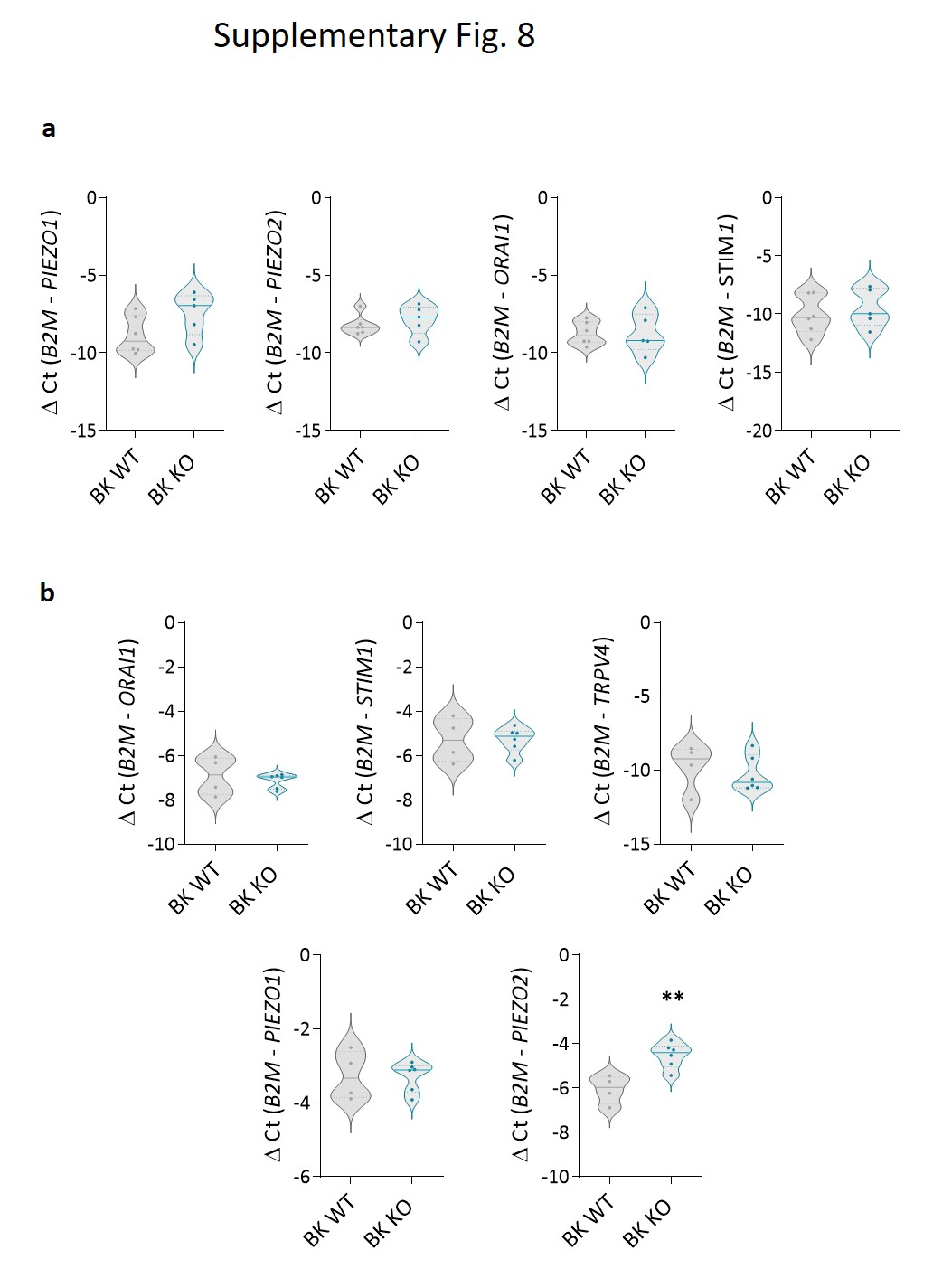


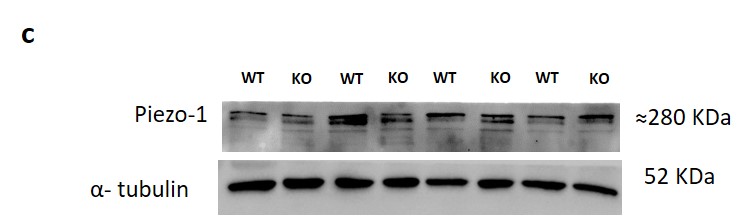


**Figure S8** **Expression of calcium influx channels in BK WT vs BK KO mice a)** qPCR showing similar mRNA expression levels of piezo-1, piezo-2, STIM and ORAI channels in BK WT (n=6) vs BK KO mice (n=5) lung homogenate. **b)** qPCR showing mRNA expression levels of piezo-1, piezo-2, STIM1, ORAI1 and TRPV4 channels in BK WT (n=4) vs BK KO mice (n=6) lung endothelial cells. **c)** Western blot analysis of protein expression of piezo-1 in BK WT (n=4) vs BK KO (n= 4) lung endothelial cells. Data are represented as violin plots showing individual values and medians.

Supplementary table 1

| Cell type | Sl. No. | Sex | mPAP (mmHg) |
| --- | --- | --- | --- |
|  |  |  |  |
| hPAEC | Donor 1 | Female |  |
| hPAEC | Donor 2 | Female |  |
| hPAEC | Donor 3 | Female |  |
| hPAEC | Donor 4 | Female |  |
| hPAEC | Donor 5 | Female |  |
| hPAEC | Donor 6 | Male |  |
| hPAEC | Donor 7 | Female |  |
| hPAEC | Donor 8 | Male |  |
| hPAEC | Donor 9 | Male |  |
| hPAEC | Donor 10 | Female |  |
| hPAEC | Donor 11 | Male |  |
| hPAEC | Donor 12 | Male |  |
|  |  |  |  |
| hPAEC | IPAH 1 | Female | 86 |
| hPAEC | IPAH 2 | Female | 82 |
| hPAEC | IPAH 3 | Female | 55 |
| hPAEC | IPAH 4 | Female | 62 |
| hPAEC | IPAH 5 | Male | 56 |
| hPAEC | IPAH 6 | Female | 71 |
| hPAEC | IPAH 7 | Male | 90 |
|  |  |  |  |
| hPASMC | Donor 13 | Female |  |
| hPASMC | Donor 14 | Female |  |
| hPASMC | Donor 15 | Female |  |
| hPASMC | Donor 16 | Male |  |
| hPASMC | Donor 17 | Female |  |
| hPASMC | Donor 18 | Male |  |
|  |  |  |  |
| hLung | Donor 19 | Male |  |
| hLung | Donor 20 | Male |  |
| hLung | Donor 21 | Female |  |
| hLung | Donor 22 | Male |  |
| hLung | Donor 23 | Male |  |
| hLung | Donor 24 | Male |  |

**Table 1:** **Cell and tissue used in the present study reported with corresponding gender of origin**
