# Supplementary material for: BK channels are indispensable for endothelial function in small pulmonary arteries
Source: Cell Commun Signal. 2025 Oct 21;23:448. doi: 10.1186/s12964-025-02436-0 (PMC12542031; doi:10.1186/s12964-025-02436-0)

**Western blot images uncropped**


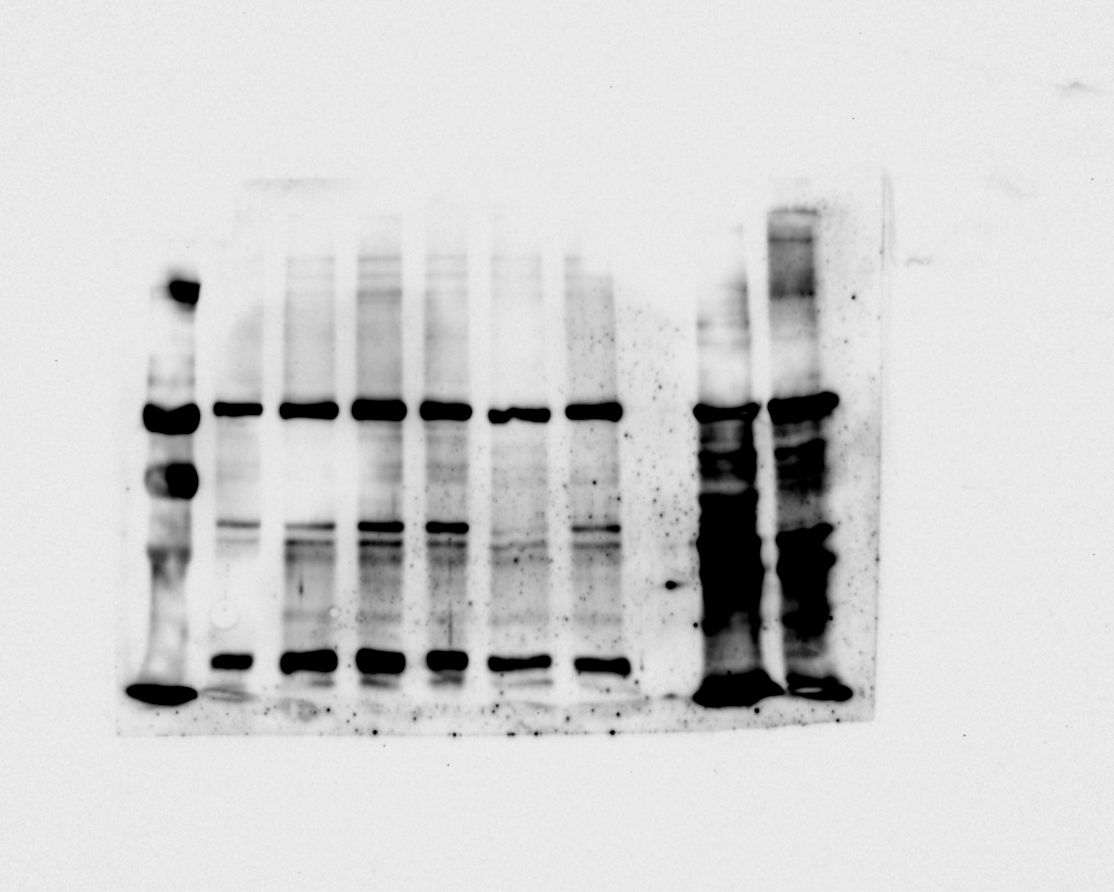


**Fig 3b BKCa**


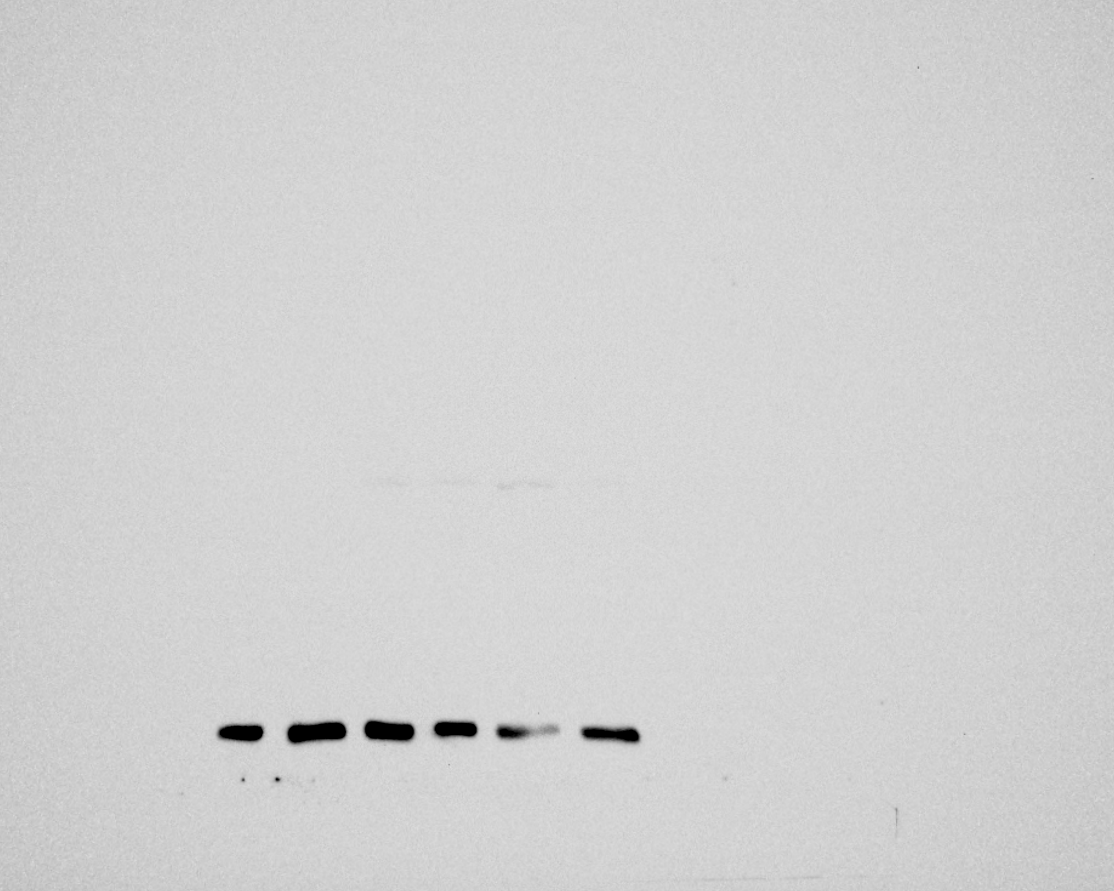


**Fig 3b GAPDH**


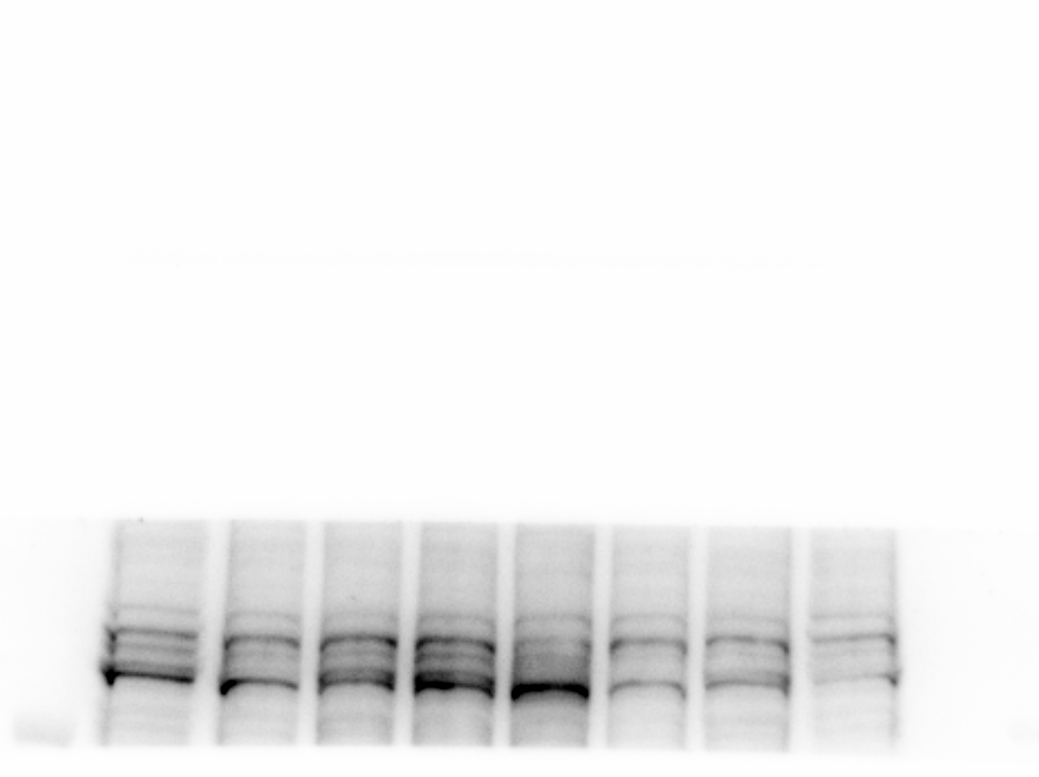


**Fig 4c BK**


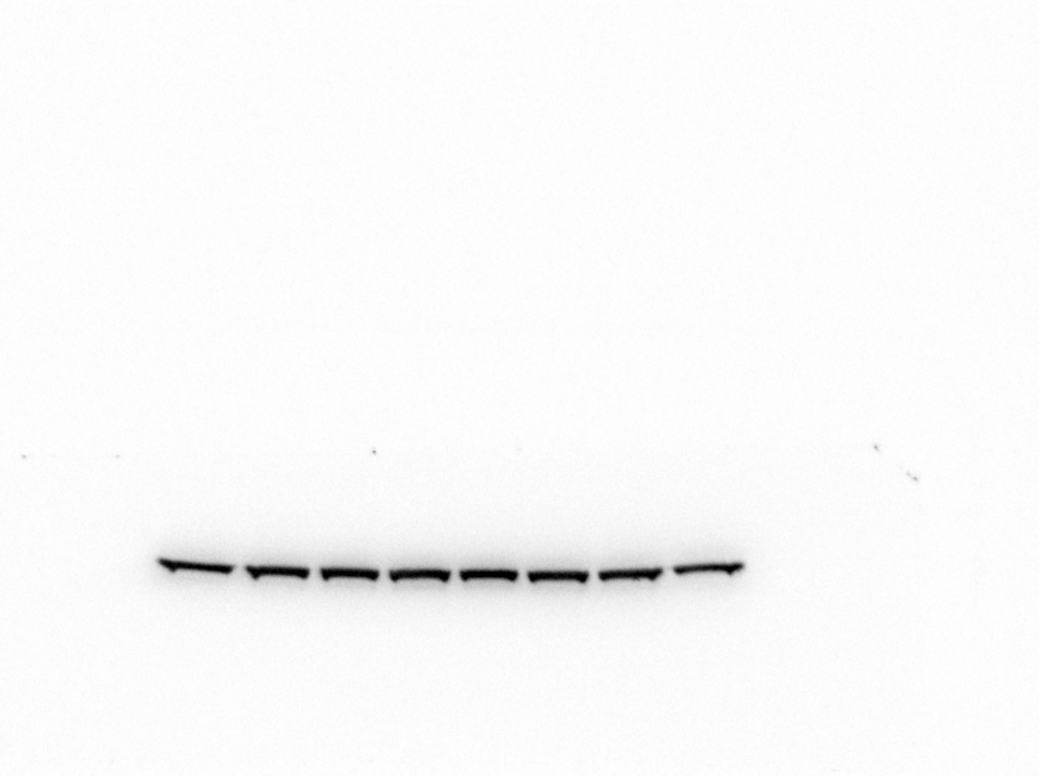


**Fig 4c B-Actin**


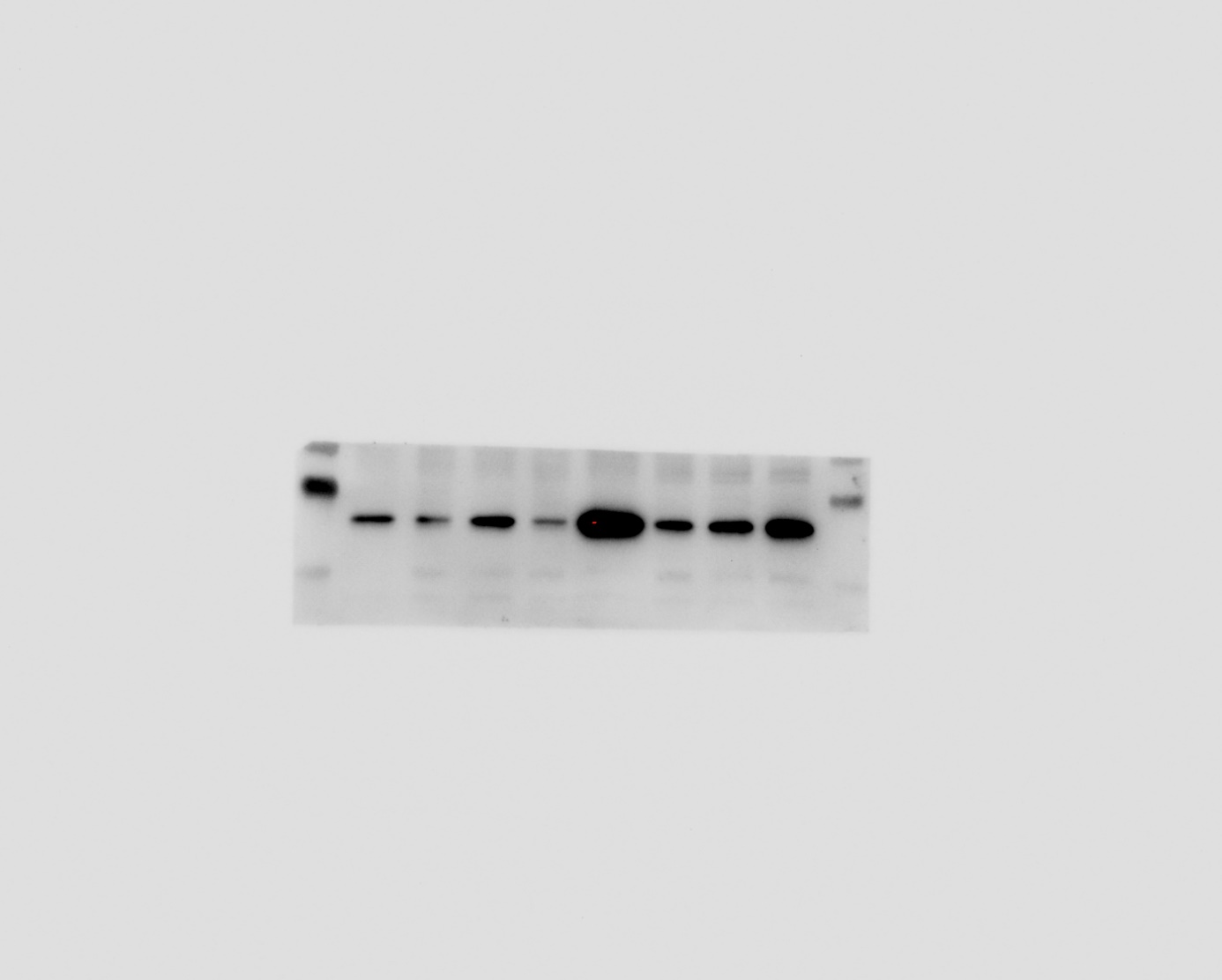


**Supplementary Fig 5b Caveolin-1**


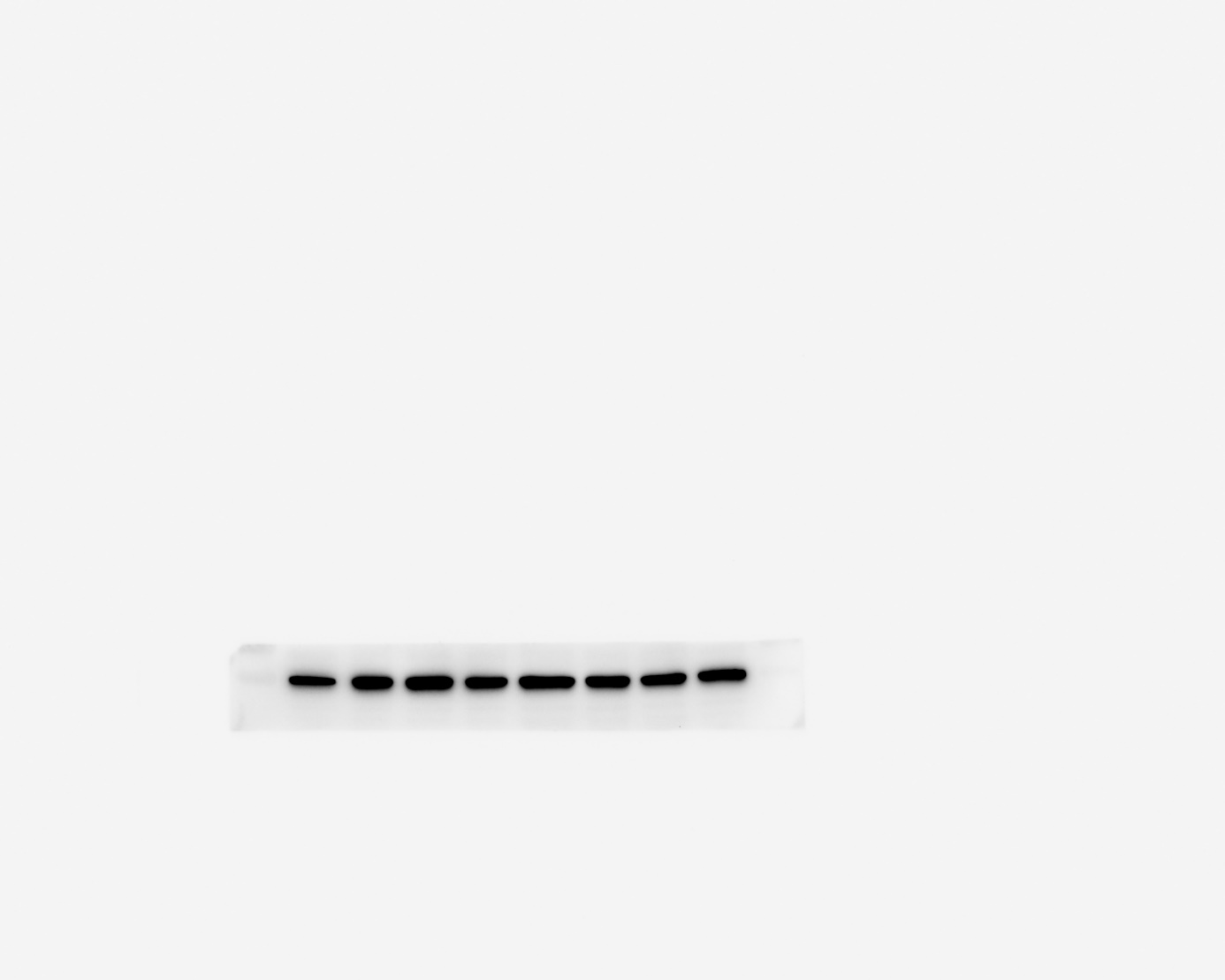


**Supplementary Fig 5b**

**α-tubulin**


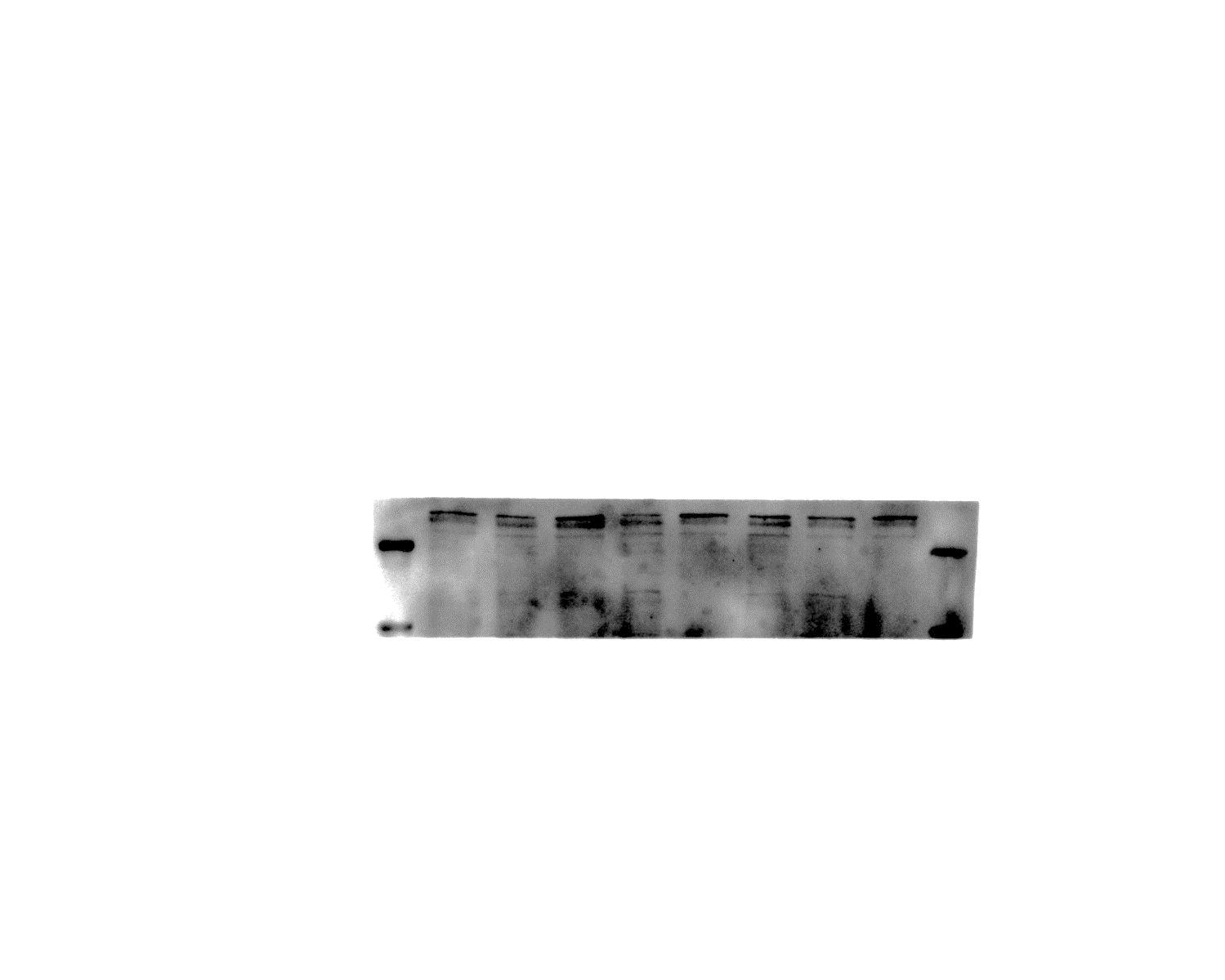


**Supplementary Fig 8c Piezo-1**

**Supplementary Fig 8c**

**α-tubulin**

**Supplementary Fig 8c α-tubulin**


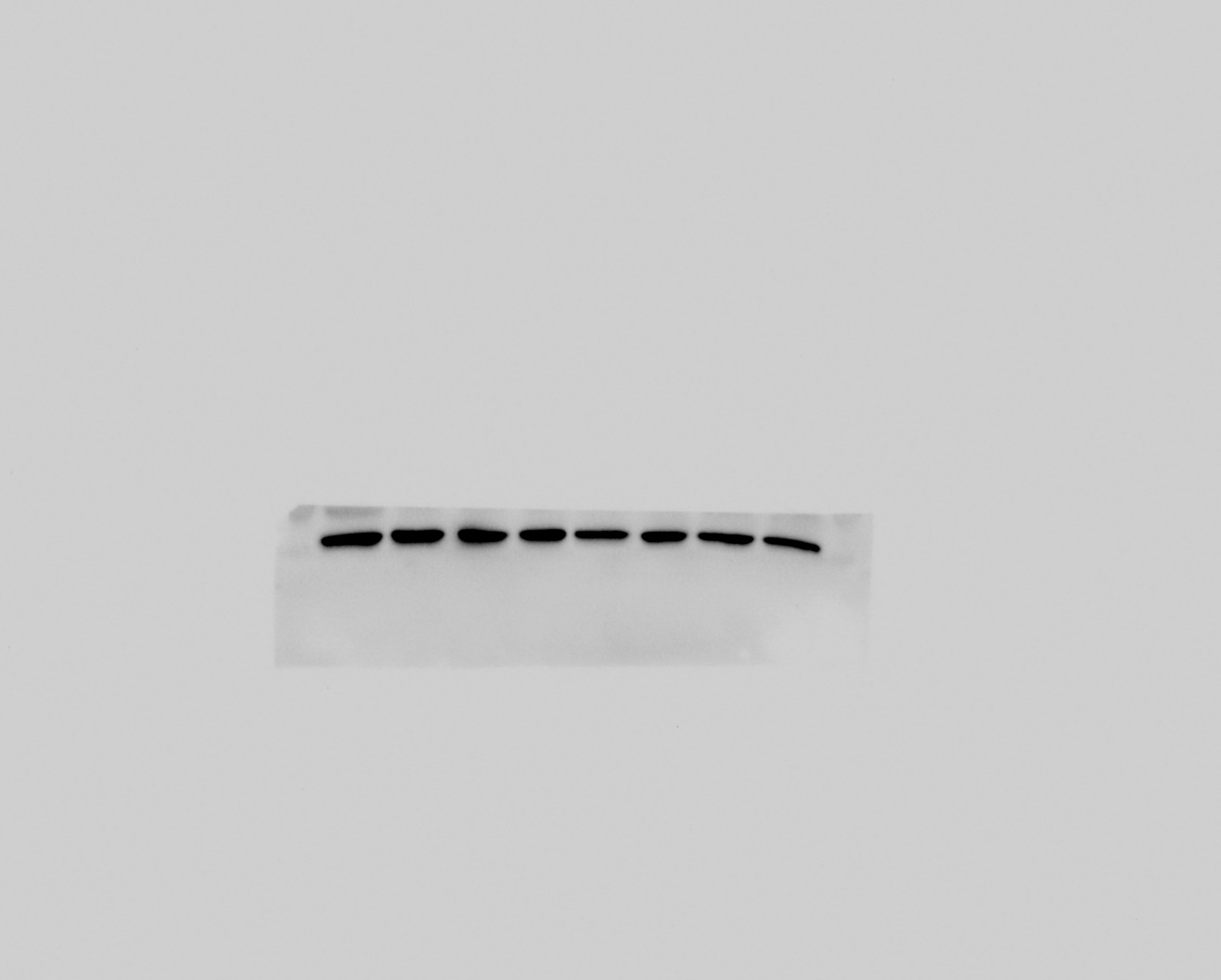

Supplement: Supplementary file 2 — Additional file 2. Uncropped Western blots. [file 12964_2025_2436_MOESM2_ESM.docx]
